# Supramolecular Self‐Assembly of β^3^‐Peptides Mediated by Janus‐Type Recognition Units

**DOI:** 10.1002/chem.202003107

**Published:** 2020-08-31

**Authors:** Selda Kabata Glowacki, Konrad Koszinowski, Dennis Hübner, Holm Frauendorf, Philipp Vana, Ulf Diederichsen

**Affiliations:** ^1^ Institute of Organic and Biomolecular Chemistry Georg-August-University Göttingen Tammannstrasse 2 37077 Göttingen Germany; ^2^ Institute of Physical Chemistry Georg-August-University Göttingen Tammannstrasse 6 37077 Göttingen Germany; ^3^ Center for Biostructural Imaging of Neurodegeneration (cfBIN) University Medical Center Göttingen von-Sieboldstrasse 3a 37075 Göttingen Germany

**Keywords:** beta-peptides, foldamers, molecular recognition, self-assembly, supramolecular chemistry

## Abstract

To gain mechanistic insights, natural systems with biochemical relevance are inspiring for the creation of new biomimetics with unique properties and functions. Despite progress in rational design and protein engineering, folding and intramolecular organization of individual components into supramolecular structures remains challenging and requires controlled methods. Foldamers, such as β‐peptides, are structurally well defined with rigid conformations and suitable for the specific arrangement of recognition units. Herein, we show the molecular arrangement and aggregation of β^3^‐peptides into a hexameric helix bundle. For this purpose, β‐amino acid side chains were modified with cyanuric acid and triamino‐*s*‐triazine as complementary recognition units. The pre‐organization of the β^3^‐peptides leads these Janus molecule pairs into a hexameric arrangement and a defined rosette nanotube by stacking. The helical conformation of the subunits was indicated by circular dichroism spectroscopy, while the supramolecular arrangement was detected by dynamic light scattering and confirmed by high‐resolution electrospray ionization mass spectrometry (ESI‐HRMS).

## Graphical abstract

**The art of directing an ensemble**: Cyanuric‐ and triamino‐*s*‐triazine‐functionalised β^3^‐peptides were designed to form stable secondary structures. Upon mixing of these pre‐organised building blocks, an intermolecular force is driven by the complementary recognition units to result in higher‐ordered structure.

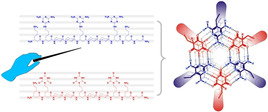

In Nature, a multitude of complex supramolecular structures consisting of limited and repeating building blocks are formed by hierarchical molecular organization, which explains how biological systems cope with the enormous workload of different tasks.^1^ Therefore, molecular self‐organization, which is triggered by intermolecular interactions and coordinative forces, is essential for living organisms. These non‐covalent interactions overcome the unfavorable entropy of the arrangement and keep an aggregate at a thermodynamic minimum, as is the case with DNA duplex formation and protein folding.^2^ Besides the understanding of the different types of well‐defined structures, the creation, control and imitation of such systems is a topic of great interest for applied science.^3^

Based on α‐peptides, synthetically produced oligomers with a characteristic tendency to fold and form stable secondary structures in solution are called foldamers.^4^ The β‐peptides belong to the most important representatives of bioinspired foldamers.^4^, ^5^, ^6^, ^7^ A chain length of six amino acids is already sufficient for stable β‐peptide secondary structures.^6^, ^8^, ^9^, ^10^ In addition, β‐peptides have a large repertoire of possible secondary structure motifs. One of the most prominent secondary structures is the 14‐helix, which has a total dipole moment inverse to an α‐helix and consists of a 14‐membered ring stabilized by hydrogen bonds to produce three flanks.^6^, ^9^, ^10^ By functionalizing the flanks of a 14‐helix with recognition units at a distance of *i* and *i+*3, the side chains of the inserted units are aligned with a stacking distance of 5 Å. The control over geometry, stoichiometry and specificity of self‐association can be increased by using specific recognition units.^11^ In previous work we could show assemblies based on the 14‐helical structure of β^3^‐peptides, using canonical nucleobases as recognition units.^12^ Complementary nucleobase‐functionalized β‐peptides could form stable helix dimers by Watson–Crick and Hoogsteen base pairing.

Driven by our previous studies and inspired by the integral membrane proteins, such as the α‐helix bundle and the β‐barrel proteins, we seek to generate an artificial pore‐forming model system with molecular self‐assembly properties of the β^3^‐peptides and corresponding recognition units (Figure 1). A well‐established recognition motif is the Janus molecule pair cyanuric acid (CYA) and triamino‐*s*‐triazine (TAT) with complementary acceptor‐donor positions.^2^, ^13^

**Figure 1:**
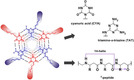
Supramolecular assembly (left side, top view) consists of the pre‐organized subunits triamino‐*s*‐triazine (blue) and cyanuric acid‐functionalized β^3^‐peptides (red). The 14‐helical conformation of the β^3^‐peptides is defined by the number of atoms in the ring formed by intramolecular hydrogen bonds between the carbonyl oxygen and the amide proton of the peptide backbone (violet arrow). The design of these peptides ensures that all CYA or TAT units are present on one side of the helix. The core consists of stacked CYA‐TAT layers connected by a network of hydrogen bonds.

Although the natural nucleobase pairing is limited to a maximum of three hydrogen bonds, the units CYA and TAT with two equivalent binding sites can interact simultaneously via N‐H⋅⋅⋅O and N‐H⋅⋅⋅N and form two times three hydrogen bonds per molecule. This interaction enables the following possible motifs: the cyclic hexamer (rosette), the linear band and the zigzag structure, which continue indefinitely.^2^, ^14^ We postulate that the pre‐organizing β^3^‐peptides CYA and TAT will align in favor of the rosette structure. The implementation of CYA and TAT molecules on β^3^‐peptides in position *i* and *i*+3 leads to stacking of rosette monolayers. Further, the specific sequential arrangement of the d‐β^3^‐amino acids (Figure 2) will enable organization of recognition units on one face of a right‐handed 14‐helix.^12^ Furthermore, the flexibility of the backbone can be minimized and a high population of the helical conformation in aqueous media can be achieved by introducing the cyclic amino acid *trans*‐2‐aminocyclohexane carboxylic acid (ACHC), which is predestined to form a 14‐helix.^15^ The Cα‐Cβ bond is fixed by the cyclohexane ring and rotation around this bond axis is prevented. Therefore, (1*R*,2*R*)‐*trans*‐ACHC‐OH improves the stability of the right‐handed helix. The solubility of decapeptides in aqueous buffers is increased by flanking two homolysines.

**Figure 2:**
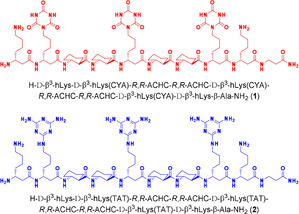
β^3^‐CYA decapeptide **1** and β^3^‐TAT decapeptide **2** prepared by solid‐phase peptide synthesis.

Initially, the two amino acids Fmoc‐d‐β^3^‐hLys(CYA)‐OH and Fmoc‐d‐β^3^‐hLys(TAT)‐OH had to be synthesized (see supporting information for experimental details). The syntheses were started with their Boc‐*N*α protected α‐amino acid analogues, which were stable against basic conditions. The key reactions involved the extension of the amino acid backbone by one methylene unit using Arndt–Eistert‐homologation, followed by a Wolff rearrangement and a palladium‐activated, carbon‐catalyzed hydrogenolysis to remove the N_ζ_ protective group of the homolysine with good yields. Subsequently, CYA and TAT were converted by a sequence of substitution and addition reactions. After final Boc‐deprotection and Fmoc‐protection, the CYA‐functionalized amino acid was realized in seven steps with a total yield of 9 % and the TAT‐functionalized amino acid in five steps and 29 % yield. Together with Fmoc‐d‐β^3^‐hLys(Boc)‐OH and Fmoc‐(1*R*,2*R*)‐*trans*‐ACHC‐OH, the artificial amino acids were used in microwave‐assisted solid phase peptide synthesis (SPPS) according to the Fmoc‐protocol (see supporting information for experimental details). Peptide synthesis was performed on a Sieber amide resin using a combination of *O*‐(7‐azabenzotriazol‐1‐yl)‐*N,N,N′,N′*‐tetramethyluronium hexafluoro‐phosphate (HATU)/7‐aza‐1‐hydroxybenzotriazole (HOAt) for producing the reactive ester from the β^3^‐amino acids in *N*,*N*‐dimethylformamide (DMF) or *N*‐methylpyrrolidin‐2‐one (NMP) by activation with *N*‐ethyl‐*N*‐(propan‐2‐yl)propan‐2‐amine (DIEA).

For all amino acids except Fmoc‐(1*R*,2*R*)‐*trans*‐ACHC‐OH a 10‐minute microwave irradiation at 50 °C and 50 W was used. In case of ACHC, a protocol was adapted by dissolving the reactants in the chaotropic salt lithium chloride (0.8 m) in NMP to prevent sequence length dependent aggregations on the resin and irradiating them for 10 minutes at 45 °C and 25 W.^16^ The performance of the second ACHC was more difficult than the first ACHC and could be optimized by extending the irradiation time to 20 minutes. For each amino acid double coupling was performed. After mild acidic cleavage from the solid support, the crude products β^3^‐CYA decapeptide **1** and β^3^‐TAT decapeptide **2** were purified by high performance liquid chromatography (HPLC) to achieve purities of ≥95 %. The integrity of the peptide sequences was confirmed by high‐resolution electrospray ionization mass spectrometry (ESI‐HRMS) (see supporting information).

Folding of peptides **1** and **2** in a 14‐helix conformation was verified by circular dichroism (CD). The CD spectra showed positive Cotton effects with maxima at 211 nm (see supporting information). The provided absorption minima below 200 nm were consistent with the reference data, nevertheless, these values were strongly influenced by the aqueous buffer.^8^, ^17^ Temperature‐dependent CD spectra were recorded at temperatures varying from 0 °C to 80 °C in 20 °C intervals. For both peptides, a decrease in molar ellipticity was visible, which was induced by thermal destabilization of the helix. These results support the formation of right‐handed 14‐helixes, as known from comparable β^3^‐peptide derivatives.^17^

To identify and characterize supramolecular arrangements, first CD and UV measurements were performed on an equimolar mixture of peptides **1** and **2** (for analytical data see supporting information). The peptide mixture yields a maximum at 212 nm with destabilization of the 14‐helix at higher temperatures. The observed spectra seem to be a direct result of the intrinsic properties of the individual components rather than the higher assemblies. To obtain further insight, concentration dependent CD and UV spectra were obtained from the 1:1 mixture of peptides **1** and **2** at concentrations of 50 μm to 500 μm. While no effect was seen immediately after mixing, significant changes were observed after a one‐day incubation period. The CD spectra showed a maximum shift of 5 nm to 206 nm and an increase in intensity at higher concentrations. The corresponding UV spectra showed a weak hyperchromic effect around 240–245 nm and a slight red shift for higher concentrations. Especially in the absorption region of the chromophores CYA and TAT, the CD spectra showed no significant changes in photophysical properties, as it is expected for a 14‐helix with aromatic units being pre‐organized with respect to the orientation of the chromophores towards the chiral helix.

Further, dynamic light scattering (DLS) was used as a non‐invasive method to determine particle size and to detect aggregates.^18^ Peptides **1**, **2** and the equimolar mixture **1**+**2** were prepared in triethylammonium acetate and incubated overnight at +4 °C. Direct conversion of the raw data, the autocorrelation curve, yielded the intensity distribution over the hydrodynamic diameter (for analytical data see supporting information). The technique is sensitive, since the ratio of the intensity of the light scattered by the particle is proportional to the sixth power of the particle diameter.^18^ Using Mie theory, we extracted volume and number particle size distributions as valuable output for the interpretation of aggregation, minimizing the intensity distortion to larger sizes. To obtain the size distributions for aggregates, the following assumptions were considered: All particles are spherical with a homogeneous and equivalent density. The intensity size distribution is correct. The optical properties of all particles in the sample are known (e.g. refractive index and viscosity at certain temperatures).^19^ Aggregate formation is identified by the comparison of particles with respect to the relative volume and number fraction (Figure 3). The peptides **1** and **2** showed a hydrodynamic diameter with a maximum below 1 nm, while the mixture **1**+**2** no longer contained individual peptides and consisted of particles with hydrodynamic sizes of 2.3–3.1 nm (number %) and 2.7–3.6 nm (for volume %) indicating the formation of expected aggregates. Increasing the concentration of peptides from 125 μm to 500 μm resulted in a shift to a smaller hydrodynamic diameter. The hexameric bundle as a non‐spherical particle should give an average value of its small side (≈1.6 nm, side view) and its long side (≈1.5–3.7 nm, top view). The particle sizes provided by DLS measurements are in agreement with the estimated length dimensions (Figure 4).

**Figure 3:**
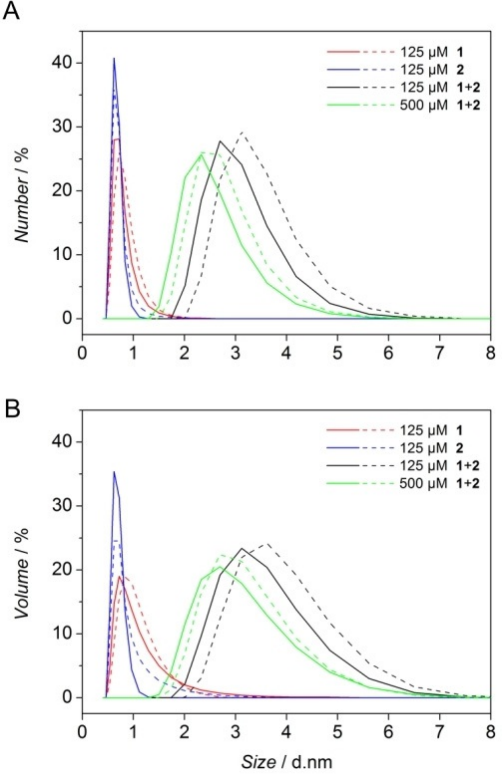
Dynamic light scattering of complementary β^3^‐decapeptides. (A) Number size distribution and (B) volume size distribution of β^3^‐CYA decapeptide **1** (red) and β^3^‐TAT decapeptide **2** (blue) and their equimolar mixture **1**+**2** (black and green) in triethylammonium acetate (5.0 mm, pH 7.4) were obtained from the first (solid line) and second measurement (dashed line).

**Figure 4:**
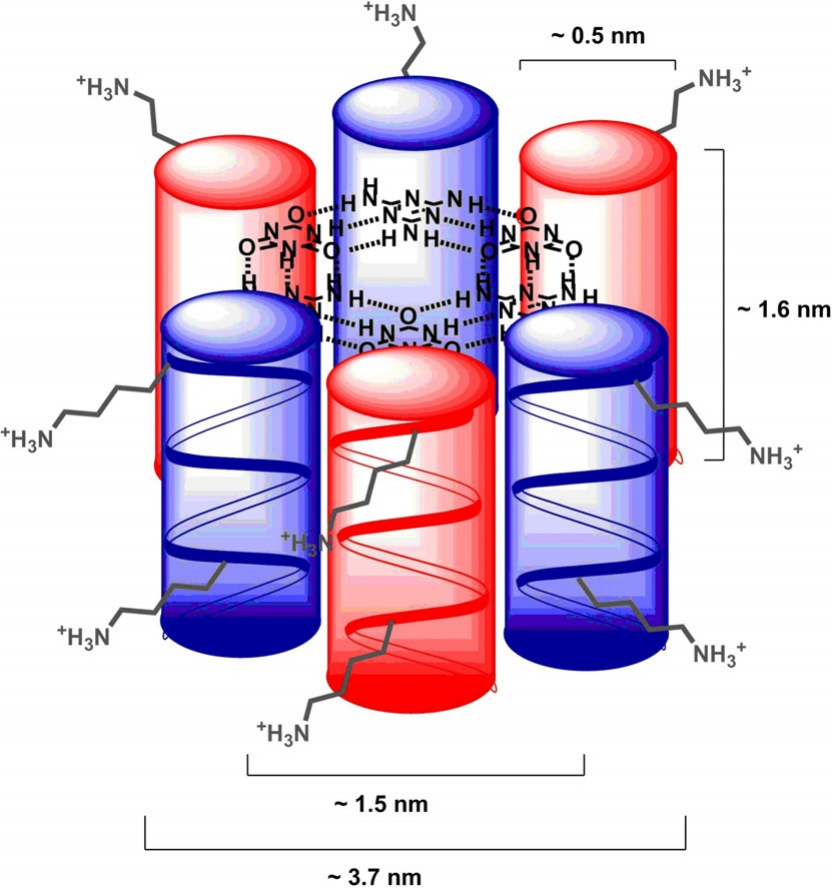
Schematic representation of the hexamers **(1**+**2)_3_** shown as a tightly packed bundle structure. Length dimensions were calculated from known values for individual parts.^6^, ^13^, ^24^ A β^3^‐peptide helix has a diameter size of 0.5 nm (top view) and a length of 1.6 nm (side view). The hexamer should result in a diameter size of 1.5 nm for the CYA‐TAT core, in a maximum diameter size of 3.7 nm (top view) and in a side length of the β^3^‐peptide helix (1.6 nm, side view).

Electrospray ionization mass spectrometry (ESI) was used to confirm the formation of higher order aggregates with an independent analytical method. Soft ionization is suitable for transfer of non‐covalent aggregates in the gas phase without fragmentation.^20^, ^21^, ^22^ The equimolar mixture **1**+**2** was prepared in the volatile ammonium acetate buffer, leading to clean mass spectra by suppressing salt adduct formation.^21^, ^23^ ESI measurements in the positive ion mode revealed the desired hexameric structure (**1**+**2**)_3_ as proton adducts (Figure 5, see Supporting Information). The charge state was determined as 7+ and 8+ by the isotope patterns with 0.14 and 0.125 *m*/*z* spacing, respectively. The calculation of the theoretical *m*/*z* ratios for ((**1**+**2**)_3_+7H)^7+^ and ((**1**+**2**)_3_+8H)^8+^ was in excellent agreement with the measured values. Besides the hexamer, the monomers of **1** and **2** were detected as di‐ and tri‐cation together with the triple‐charged heterodimer ((**1**+**2**)+3H)^3+^ as the major fragments. The assignment was additionally supported by collision‐induced dissociation (CID) experiments, which showed a voltage‐dependent dissociation of the hexamer into single and double charged monomers and triple charged heterodimers only within the analyzed range.

**Figure 5:**
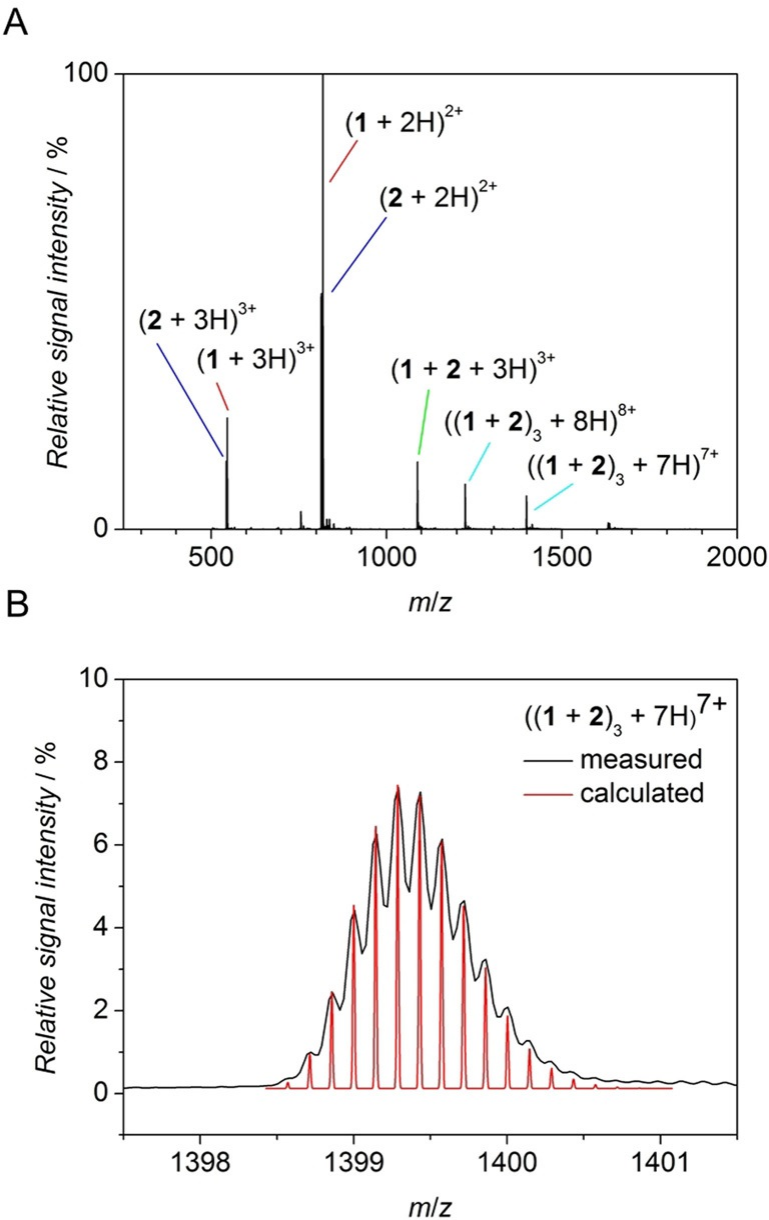
Mass analysis of complementary β^3^‐peptides. (A) Positive ion mode ESI‐mass spectrum of a solution of an equimolar mixture of β^3^‐CYA decapeptide **1** and β^3^‐TAT decapeptide **2** (50 μm in 5 mm ammonium acetate diluted prior analysis to 20 μm final peptide concentration). (B) Isotope pattern of desired hexameric aggregate **(1**+**2)_3_** measured (black) and calculated (red).

Interestingly, apart from the heterogeneous dimer and hexamer, no further intermediate oligomers such as trimer, tetramer or pentamer could be observed in all ESI and CID experiments above an analyzed relative signal intensity of 0.5 %. After formation of the dimer, the driving force is high for generating the hexamer aggregate due to the favorable pre‐organization of the recognition units CYA and TAT and the high stability of the hydrogen bonded network.

In conclusion, the design and synthesis of an artificial two‐component system consisting of cyanuric acid and triamino‐*s*‐triazine‐functionalized β^3^‐peptides was presented. ESI‐MS showed the mass of the expected structure with hexameric topology, whereas CID verified the highly selective formation of this non‐covalent structure. Full consumption of the starting materials was indicated in DLS with promising results corresponding to the expected particle diameter range of the assembly. In addition, concentration and time‐dependent UV and CD spectra, as well as temperature‐dependent CD spectra, were recorded with hardly noticeable changes, indicating the presence of soluble aggregates. Overall, we can conclude on the presence of a distinct hexameric β‐peptide helical bundle, which was formed by organization at the nanomolecular level. These results underline the potential of the helix bundle for further investigation by extending its length to fit into a model membrane. The extended transmembrane helix bundle will open up biochemical and biophysical studies, in which the ability to act as a transmembrane helix bundle protein and to transport ions or water will be investigated.

## Conflict of interest

The authors declare no conflict of interest.

## Supporting information

As a service to our authors and readers, this journal provides supporting information supplied by the authors. Such materials are peer reviewed and may be re‐organized for online delivery, but are not copy‐edited or typeset. Technical support issues arising from supporting information (other than missing files) should be addressed to the authors.

Supplementary
