# Supplementary material for: Supramolecular Self‐Assembly of β3‐Peptides Mediated by Janus‐Type Recognition Units
Source: Chemistry. 2020 Aug 31;26(53):12145–9. doi: 10.1002/chem.202003107 (PMC7539953; doi:10.1002/chem.202003107)
Supplement: Supplementary file 1 — Supplementary [file CHEM-26-12145-s001.pdf]

Chemistry–A European Journal

Supporting Information

Supramolecular Self-Assembly of β³-Peptides Mediated by Janus-Type Recognition Units

Selda Kabata Glowacki,[a, c] Konrad Koszinowski,[a] Dennis Hübner,[b] Holm Frauendorf,[a] Philipp Vana,[b] and Ulf Diederichsen*[a]

Table of Contents

1. Chemicals, Materials and Instrumentation S2
2. β^3-D-Amino Acids S3
   2.1 Synthetic Scheme S3
   2.2 General Synthetic Procedure S4
   2.3 Synthesis and Characterization S4
3. β^3-D-Peptides S9
   3.1 Synthetic Scheme S9
   3.2 General Synthetic Procedure S9
   3.3 Synthesis and Characterization S11
4. Aggregation Studies S11
   4.1 Circular Dichroism (CD) and Ultraviolet (UV) Spectroscopy S12
   4.2 Dynamic Light Scattering (DLS) S14
   4.3 Electrospray-Ionization (ESI) Mass Spectrometry S15
5. Supporting Figures and Spectra S18
   5.1 ^{1}H- and ^{13}C-NMR-Spectra of β^3-D-Amino Acids S18
   5.2 HPLC-Chromatograms and ESI-HRMS Spectra of β^3-D-Peptides S27
6. References S29

1. Chemicals, Materials and Instrumentation

Reagents. Starting materials and reagents were of the highest grade available from commercial sources and were used as delivered. The chemicals for peptide synthesis, amino acids, coupling reagents and resin, were purchased from IRIS BIOTECH (Marktredwitz, Germany), CARL ROTH (Karlsruhe, Germany) BACHEM (Bubendorf, Switzerland), GL BIOCHEM (Shangai, China), and NOVABIOCHEM (Merck Millipore, Darmstadt, Germany). Flash column chromatography on silica was performed using MACHEREY-NAGEL Silica Gel 60 with a particle size of 0.063–0.2 mm (Düren, Germany). Thin layer chromatography (TLC) was conducted on aluminum backed plates of silica gel 60 F254 (layer thickness: 0.20 mm) from MERCK (Darmstadt, Germany) to detect UV-active spots by fluorescence quenching at 254 nm and 366 nm. Non-fluorescence quenching substances were visualized by staining with 3% ninhydrin and acetic acid (AcOH) in ethanol (EtOH) (*w*/*v*/*v*) and subsequent careful heat-drying.

Solvents. Anhydrous solvents of extra dry or puriss. absolute grade (over molecular sieves) were obtained from ACROS-ORGANICS (Geel, Belgium) and MERCK (former Sigma Aldrich, Schnelldorf, Germany). Deuterated solvents were purchased from DEUTERO (Kastellaun, Germany). Technical solvents were distilled prior to use in flash column chromatography. Acetonitrile (CH_3CN) and methanol (MeOH) for high performance liquid chromatography (HPLC) in respective grade and all other solvents of the grade puriss. p.a. were purchased from commercially available sources and were used as supplied. Water for HPLC (milliQ H_2O) and buffers was purified using the ultrapure water unit Simplicity from MERCK MILLIPORE (Darmstadt, Germany). Buffers were degassed by stirring under vacuum and kept under argon atmosphere afterwards. The pH of buffers was adjusted before every use.

Reactions. All air- and water-sensitive reactions were conducted under inert gas atmosphere (argon or nitrogen). Therefore, glass equipment utilized for large scale reactions was flame-dried before use. A purge-and-refill technique was applied for small reactions, which were performed in non-heatable tubes.

Instruments. ^{1}H-, ^{13}C-nuclear magnetic resonance (**NMR**) spectra were performed using a Avance III 400 (AV-401) instrument from BRUKER (Billerica, Massachusetts, USA) or Mercury (VX) 300, a Unity 300 and a Inova-500 instruments from VARIAN (California, USA). Chemical shifts are quoted in ppm (TMS = 0 ppm). The resonances of the rest protons of deuterated solvents were taken as internal standards. ^{13}C-NMR spectra were recorded as broadband ^{1}H-decoupled or APT spectra. Multiplicities are abbreviated as follows: s = singlet, d = doublet, t = triplet, q = quartet, m = multiplet, br = broad, dd = doublet of doublets, dt = doublet of triplets, dq = doublet of quartets, ddd = doublet of doublet of doublets. Coupling constants $^{n}J_{x,x}$ are stated in Hz, n is the number of bonds mediating the coupling, x stands for the nuclei. [^{1}H-^{1}H]-COSY-, HSQC- und HMBC-experiments were performed to assign the signals to the respective atoms of analyzed compound. High-resolution electrospray ionization (**ESI-HRMS**) mass spectra were recorded with a maXis ESI-QTOF-MS and a micrOTOF-Q II spectrometer from BRUKER (Bremen, Germany). The values are given as mass per charge (*m/z)*. Microwave-mediated solid phase peptide synthesis (**SPPS**) was realized on a DiscoverSPS microwave-synthesizer from CEM (Kamp-Lintfort, Germany). Precipitates from post-cleavage work-up were processed on a centrifuge from SIGMA 2K15C (Osterode am Harz, Germany). Reverse-phase high-performance liquid chromatography (**HPLC**) was performed on a Pharmacia Äkta Basic system from GE HEALTHCARE (London, UK) with a pump-type P-903, and a variable wavelength detector ultraviolet (UV)-900 using a MACHEREY-NAGEL Nucleodur® RP C-18 analytical HPLC column (250 × 4.6 mm, 5 µm) and a semipreparative HPLC column (250 × 10 mm, 5 µm). The HPLC runs were carried out using a linear gradient of 0.1% aq. TFA (solvent A) and 80% aq. CH_3CN/0.1% TFA (solvent B) in 30 min. Flow rates were taken as 1 mL/min for the analytical, and 3 mL/min for the semi-preparative purpose. The UV absorbance of peptides could be detected at three different wavelengths (215 nm, 254 nm and 280 nm) simultaneously. Freeze-drying of compounds from aqueous solutions containing minimal amounts of MeOH or acetonitrile was performed using a Christ-Alpha-2-4 **lyophilizer** attached to a high vacuum pump and a CHRIST RCV-2-18 ultracentrifuge (Osterode am Harz, Germany). UV-spectra were recorded on a V-550 UV/vis- or V-650 **UV/vis spectrophotometer** from JASCO (Groß-Umstadt, Germany). Circular dichroism (CD) spectra were performed on a J840-A **CD spectrophotometer** from JASCO (Groß-Umstadt, Germany). The quartz glass cuvette Suprasil® (QS) were supplied by HELLMA (Müllheim, Germany). The shown UV- and CD-spectra were displayed with SAVITZKY-GOLAY filter. Dynamic light scattering (**DLS**) was performed on Zetasizer Nano S equipped with a standard laser at 633 nm from MALVERN PANALYTICAL (Malvern, United Kingdom).

Softwares. ChemBioDraw from PERKIN ELMER (Waltham, Massachusetts, USA) was utilized to draw chemical structures. Graphs were plotted with the program OriginPro 8.5 from ORIGINLAB CORPORATION (Northampton, Massachusetts, USA) and NMR-spectra were analyzed with MestReNova 7 from MESTRELAB RESEARCH (Santiago de Compostela, A Coruña, Spain).

2. β³-D-Amino Acids

2.1 Synthetic Scheme

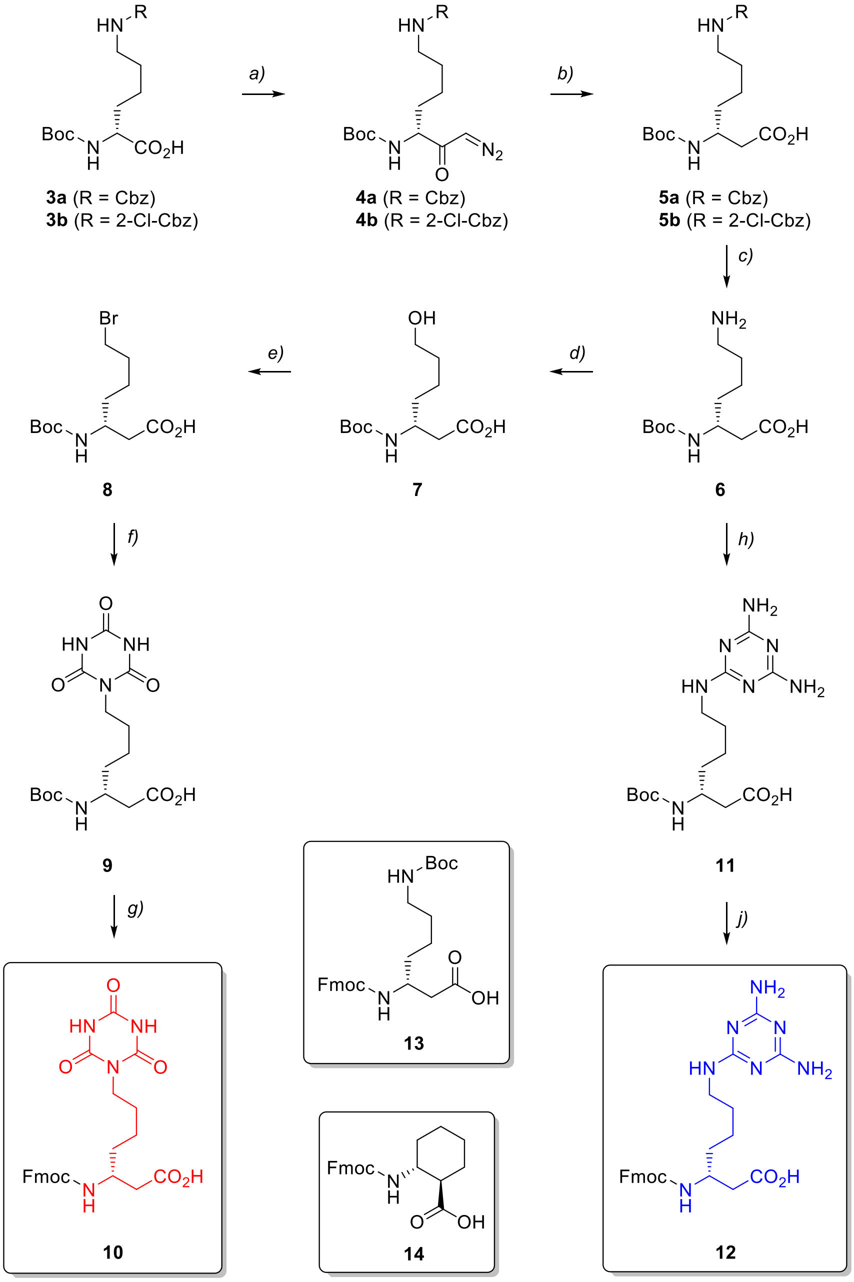

Scheme S1. Overview of unnatural amino acids used for peptide synthesis (framed compounds **10**, **12**, **13** and **14**) and the synthetic routes for Fmoc-β³-D-hLys(CYA)-OH (**10**) and Fmoc-β³-D-hLys(TAT)-OH (**12**). For R = H a) (i) NEt_3, ^{i}BuCOOCl, THF, −21 °C, 45 min, (ii) CH_2N_2, Et_2O, 0 °C, 30 min, RT, 5 h; b) AgOBz, THF/H_2O (9:1), ultrasonication, 3 h; 84% in two steps. For R = 2-Cl-Cbz a) (i) NEt_3, ^{i}BuCOOCl, THF, -15 °C, 45 min, (ii) CH_2N_2, Et_2O, 0 °C, 30 min, RT, 6 h; 97%. b) AgOBn, THF/H_2O (9:1), ultrasonification, 4.5 h; 85%. c) Pd/C, H_2, ethanol (EtOH), RT, overnight; 98%–quant. d) $Na_2[Fe(CN)_5NO]\cdot 2H_2O$, 4 M aq. NaOH, H_2O, 65 °C, 5 h; 50%. e) PPh_3, CBr_4, CH_2Cl_2, −10 °C–0 °C, 1 h, RT, overnight; 45%. f) cyanuric acid, DBU, DMF, 70 °C, 2 d; 52%. g) (i) TFA/H_2O (95:5), 0 °C, 1 h, RT, 1 h, (ii) Fmoc-OSu, $NaHCO_3$, dioxane/H_2O (1:1), 0 °C, 1 h, RT, overnight; 90%. h) 6-Chloro-1,3,5-triazine-2,4-diamine, K_2CO_3, DMSO, 84–98 °C, overnight; 70%. j) (i) TFA/H_2O (95:5), 0 °C, 1 h, RT, 1 h, (ii) Fmoc-OSu, $NaHCO_3$, dioxane/H_2O (1:1), 0 °C, 1 h, RT, overnight; 64%.

2.2 General Synthetic Procedure

GSP 1: Synthesis of diazoketone

Under inert gas atmosphere, a solution of the amino acid (1.00 eq) in anhydrous tetrahydrofuran (THF) (~10 mL/g amino acid) was cooled to –21 °C. After addition of triethylamine (NEt_3, 1.10 eq) and isobutylchloroformate (1.10 eq) the reaction mixture was stirred 45 min at –21 °C to –15 °C. Then, the reaction mixture was warmed up to 0 °C and diazomethane[1] in dieethylether (Et_2O, 0.6–0.7 M, 2.00 eq) was added under light exclusion. The solution was stirred first for 0.5 h at 0 °C, then 4–6 h at room temperature (RT) and quenched afterwards with AcOH (2.00 eq). Et_2O (~10 mL/g amino acid) and sat. $NaHCO_3$-solution (~10 mL/g amino acid) or a 6% aq. $NaHCO_3$-solution [~10 mL/g per 9-fluorenylmethoxycarbonyl (Fmoc)-protected amino acid] was given and the layers were separated. The organic layer was washed with sat. NH_4Cl-solution (~10 mL/g amino acid) and sat. NaCl-solution (~10 mL/g amino acid), dried over $MgSO_4$ and the solvent was removed *in vacuo*. The product was immediately used for the next step or purified via flash column chromatography.

GSP 2: Synthesis of β^3-amino acids

A solution of the diazoketone from GSP 1 (1.00 eq) in THF/H_2O (9:1, *v*/*v*) was first cooled to 0 °C and then treated with silver benzoate (0.10 eq) excluding light and ultrasonicated for 1.5–4.5 h. The reaction was followed by TLC and diluted after full conversion with H_2O (~10 mL/g amino acid). The pH was adjusted with 1 M aq. HCl to 2 and Et_2O or ethyl acetate (EtOAc, ~10 mL/g amino acid) was added. The layers were separated and the aqueous layer was extracted with Et_2O or EtOAc (3 × ~10 mL/g amino acid). The combined organic extracts were dried over $MgSO_4$ and the solvent was removed under vacuum. The crude product was purified by flash column chromatography.

2.3 Synthesis and Characterization

Fmoc-(1*R*,2*R*)-*trans*-ACHC-OH (**13**) was synthesized according to procedure described by GELLMAN[2] or was commercially purchased from POLYPEPTIDE GROUP (Straßburg, France). The synthesis of Boc-β^3-D-hLys(Cbz)-OH (**5a**) and Fmoc-β^3-D-hLys(Boc)-OH (**13**) were adapted from SEEBACH[3] and DIEDERICHSEN[4] applying GSP 1 and GSP 2.

(4b) **(*R*)-3-((*tert*-Butoxycarbonyl)-amino)-7-((((2-chlorobenzyl)-oxy)-carbonyl)-amino)-1-diazoheptane-2-on, Boc-D-Lys(2-Cl-Cbz)-CHN$_2$**

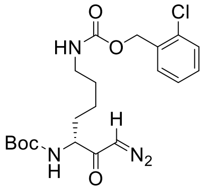

$C_{20}H_{27}ClN_4O_5$ [438.91]

Starting from Boc-D-Lys(2-Cl-Cbz) -OH (**3b**) (5.00 g, 12.1 mmol, 1.00 eq), the synthesis of compound **51** was carried out according to GSP 1. The crude product was purified by column chromatography using the eluents pentane/EtOAc (3:2, *v*/*v*) to give the product **4b** (5.14 g, 11.7 mmol, 97%) as a yellow oil. **TLC** (pentane/EtOAc, 3:2, *v/v*): R_f = 0.25. **^{1}H-NMR** (300 MHz, [D$_6$]DMSO, 308 K): δ = 1.39 (s, 9 H, $C(CH_3)_3$), 1.22–1.68 (m, 6 H, β-H_2, γ-H_2, δ-H_2), 2.96–3.02 (m, 2 H, ε-H_2), 3.80–3.92 (m, 1 H, α-H), 5.09 (s, 2 H, C<u>H</u>$_2$Ph), 6.00 (s, 1 H, CHN_2), 7.13 (d, ^3J = 7.7 Hz, 1 H, NH-Boc), 7.28 (t, ^3J = 5.5 Hz, 1 H, NH-Cbz), 7.34–7.49 (m, 4 H, 4 x Ph-H) ppm. **^{13}C-NMR** (125 MHz, [D$_6$]DMSO, 308 K): δ = 28.09 (C(<u>C</u>H$_3$)$_3$), 22.66, 28.90, 30.29 (β-CH_2, γ-CH_2, δ-CH_2), 39.95 (ε-CH_2), 52.34 (CHN_2), 58.18 (α-CH), 62.45 (<u>C</u>H$_2$Ph), 78.17 (<u>C</u>(CH$_3$)$_3$), 127.14, 127.51, 129.57 (4 × Ph-CH), 132.54, 134.54 (Ph-C), 155.35, 155.70 (Boc-<u>C</u>O, Cbz-<u>C</u>O), 195.63 (<u>C</u>OCHN$_2$) ppm. **MS** (ESI): *m/z* = 461.2 [*M* + Na]$^+$, 899.3 [2*M* + Na]$^+$, 437.2 [*M* – H]$^-$. **HR-MS** (ESI): $C_{20}H_{27}ClN_4O_5$ calculated 439.1743 [*M* + H]$^+$, 461.1562 [*M* + Na]$^+$, 437.1597 [*M* – H]$^-$, found 439.1731 [*M* + H]$^+$, 461.1553 [*M* + Na]$^+$, 437.1587 [*M* – H]$^-$.

(5b) (*R*)-3-((*tert*-Butoxycarbonyl)amino)-7-((((2-chlorobenzyl)oxy)carbonyl)amino)heptanoic acid, Boc-D-β³-hLys(2-Cl-Cbz)-OH

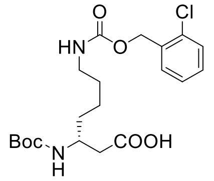

$C_{20}H_{29}ClN_2O_6$ [428.91]

Starting from Boc-D-Lys(2-Cl-Cbz)-CHN₂ (**4b**) (5.11 g, 11.7 mmol, 1.00 eq), the synthesis of compound **5b** was carried out according to GSP 2. The crude product was purified by column chromatography using the eluents DCM/MeOH (9:1→9:1+0.5% AcOH, *v*/*v*/*v*). AcOH was removed as an azeotropic mixture with toluene (3 x 20 mL) to provide the product **5b** (4.26 g, 9.94 mmol, 85%) as a viscous, light yellow oil. **TLC** (DCM/MeOH, 9:1, *v/v*): R_f = 0.45. **¹H-NMR** (300 MHz, [D₆]DMSO, 308 K): *δ* = 1.37 (s, 9 H, C(CH₃)₃), 1.14–1.46 (m, 6 H, γ-H₂, δ-H₂, ε-H₂), 2.21–2.37 (m, 2 H, α-H₂), 2.94–3.02 (m, 2 H, ζ-H₂), 3.63–3.75 (m, 1 H, β-H), 5.08 (s, 2 H, C<u>H</u>₂Ph), 6.60 (d, ^3J = 8.8 Hz, 1 H, NH-Boc), 7.24–7.27 (m, 1 H, NH-Cbz), 7.31–7.49 (m, 4 H, 4 × Ph-H) ppm. **¹³C-NMR** (125 MHz, [D₆]DMSO, 308 K): *δ* = 28.17 (C(<u>C</u>H₃)₃), 22.69, 29.09, 33.90 (γ-CH₂, δ-CH₂, ε-CH₂), 39.93, 40.21 (α-CH₂, ζ-CH₂), 47.28 (β-CH), 62.42 (<u>C</u>H₂Ph), 77.31 (<u>C</u>(CH₃)₃), 127.17, 129.14, 129.50, 129.55 (4 × Ph-CH), 132.17, 134.57 (Ph-C), 154.97, 155.68 (Boc-<u>C</u>O, Cbz-<u>C</u>O), 172.71 (COOH) ppm. **MS** (ESI): *m/z* = 451.2 [*M* + Na]⁺, 879.3 [2*M* + Na]⁺, 427.2 [*M* – H]⁻, 855.3 [*M* – H]⁻. **HR-MS** (ESI): $C_{20}H_{29}ClN_2O_6$ calculated 429.1787 [*M* + H]⁺, 451.1606 [*M* + Na]⁺, 427.1641 [*M* – H]⁻, found 429.1778 [*M* + H]⁺, 451.1610 [*M* + Na]⁺, 427.1640 [*M* – H]⁻.

(6) (*R*)-7-Amino-3-((*tert*-butoxycarbonyl)amino)heptanoic acid, Boc-D-β³-hLys-OH

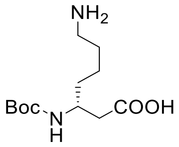

$C_{12}H_{24}N_2O_4$ [260.33]

Variant 1: Boc-D-β³-hLys(Cbz)-OH (**3b**) (6.03 g, 15.3 mmol, 1.00 eq) was dissolved in EtOH (100 mL). After degassing the solution, Pd/C (5%, 1.64 g) was added and the reaction mixture was stirred under H_2 atmosphere for 16.5 h at RT. Pd/C was filtered off and the solvent was removed *in vacuo* to led to the pure product **6** (3.90 g, 15.0 mmol, 98%) as a white powder.
Variant 2: Boc-D-β³-hLys(2-Cl-Cbz)-OH (**3b**) (7.94 g, 18.5 mmol, 1.00 eq) was dissolved in EtOH (120 mL). After degassing the solution, Pd/C (5%, 1.50 g) was added and the mixture was stirred at RT for 19.5 h under H_2 atmosphere. Pd/C was then filtered off and the solvent was removed under vacuum. The residual was purified by column chromatography on RP gel (H_2O →H_2O/MeOH, 1:1, *v*/*v*) to give the product **6** (4.65 g, 17.8 mmol, 97%) as a white powder.
TLC (EtOAc/MeOH/H₂O/AcOH, 6:2:2:1 + NaCl, *v*/*v*/*v*/*v*): R_f = 0.15. **¹H-NMR** (300 MHz, [D₆]DMSO, 308 K): δ = 1.37 (s, 9 H, C(CH₃)₃), 1.20–1.63 (m, 6 H, γ-H₂, δ-H₂, ε-H₂), 2.22–2.38 (m, 2 H, α-H₂), 2.69–2.77 (m, 2 H, ζ-H₂), 3.64–3.77 (m, 1 H, β-H), 6.65 (d, 3J = 8.7 Hz, 1 H, Boc-NH) ppm. **¹³C-NMR** (125 MHz, [D₆]DMSO, 308 K): δ = 28.20 (C(CH₃)₃), 22.40, 26.65, 33.58 (γ-CH₂, δ-CH₂, ε-CH₂), 38.50 (α-CH₂), 38.50–39.99 (ζ-CH₂ covered from [D6]DMSO signal), 47.07 (β-CH), 77.32 (C(CH₃)₃), 154.81 (Boc-CO), 172.45 (COOH) ppm. **MS** (ESI): m/z = 261.2 [*M* + H]⁺, 521.3 [2*M* + H]⁺, 295.1 [*M* – H]⁻, 519.3 [2*M* – H]⁻. **HR-MS** (ESI): $C_{12}H_{24}N_2O_4$ calculated 261.1809 [*M* + H]⁺, 283.1628 [M + Na]⁺, 259.1663 [*M* – H]⁻, found 261.1809 [*M* + H]⁺, 283.1624 [*M* + Na]⁺, 259.1663 [*M* – H]⁻.

(7) (*R*)-3-((*tert*-Butoxycarbonyl)amino)-7-hydroxyheptanoic acid

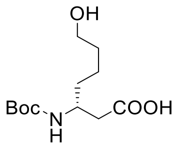

$C_{12}H_{23}NO_5$ [261.32]

Boc-D-β^3-hLys-OH (**6**) (3.86 g, 14.8 mmol, 1.00 eq) was suspended in H_2O (57 mL) and an aqueous 4 M NaOH solution (5.72 mL) was added at 60 °C. Sodium pentacyano-nitrosyl ferrate (II) dihydrate (6.75 g, 22.6 mmol, 1.53 eq) was added in smaller portions over 1 h, while the pH was adjusted to 9.5 by adding further aqueous 4 M NaOH solution (5.72 mL) to keep the pH. The mixture was stirred at 60 °C for additional 5 h. The reaction mixture was then cooled to 10 °C, and the pH was adjusted to 1 with an aqueous 1 M HCl solution. The resulting solution was extracted with EtOAc (4 × 100 mL), dried over $MgSO_4$ and the combined extracts were adsorbed on silica gel. The crude product **7** was purified by flash column chromatography using the eluents DCM/MeOH (24:1 + 0.25% AcOH → 9:1 + 1% AcOH, *v*/*v*). AcOH was removed as an azeotropic mixture with toluene (3 x 20 mL). After drying *in vacuo* and lyophilization, the pure product **7** (1.92 g, 7.34 mmol, 50%[1]) was isolated in form of a light yellow oil. **TLC** (DCM/MeOH, 9:1+1% AcOH, *v*/*v*/*v*): R_f = 0.43. **^{1}H-NMR** (300 MHz, [D$_6$]DMSO, 308 K): δ = 1.37 (s, 9 H, $C(CH_3)_3$), 1.13–1.49 (m, 6 H, γ-H$_2$, δ-H$_2$, ε-H$_2$), 2.21–2.36 (m, 2 H, α-H$_2$), 3.36 (t, ^3J = 6.4 Hz, 2 H, ζ-H$_2$), 3.64–3.76 (m, 1 H, β-H), 6.58 (d, ^3J = 8.7 Hz, 1 H, NH-Boc) ppm. **^{13}C-NMR** (125 MHz, [D$_6$]DMSO, 308 K): δ = 28.20 (C($\underline{C}H_3)_3$), 21.97, 32.26, 34.14 (γ-CH$_2$, δ-CH$_2$, ε-CH$_2$), 39.92 (α-CH$_2$), 47.33 (β-CH), 60.61 (ζ-CH$_2$), 77.32 ($\underline{C}(CH_3)_3$), 154.99 (Boc-$\underline{C}$O), 172.55 (COOH) ppm. **MS** (ESI): *m/z* = 284.2 [*M* + Na]$^+$, 545.3 [2*M* + Na]$^+$, 260.2 [*M* – H]$^-$, 521.3 [2*M* – H]$^-$. **HR-MS** (ESI): $C_{12}H_{23}NO_5$ calculated 284.1468 [*M* + Na]$^+$, 260.1503 [*M* – H]$^-$, found 284.1473 [*M* + Na]$^+$, 260.1506 [*M* – H]$^-$.

(8) (*R*)-7-Bromo-3-((*tert*-butoxycarbonyl)amino)heptanoic acid

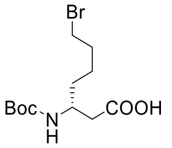

$C_{12}H_{22}BrNO_4$ [324.21]

Under inert gas atmosphere and vigorous stirring, a solution of (*R*)-3-((*tert*-butoxycarbonyl)amino)-7-hydroxyheptanoic acid (**7**) (1.87 g, 7.16 mmol, 1.00 eq) and tetrabromomethane (3.56 g, 10.7 mmol, 1.50 eq) in dry DCM (40 mL) was treated at -10 °C with triphenylphosphine (2.82 g, 10.7 mmol, 1.50 eq). After 30 min the solution was warmed up to room temperature and stirred overnight. The reaction solution was then adsorbed on silica gel and purified by column chromatography using the eluents DCM/MeOH (24:1, *v*/*v*) to led to the product **8** (1.03 g, 3.19 mmol, 45%[2]) as a light yellow oil, which becomes a white solid after storage at −21 °C. **TLC** (DCM/MeOH, 24:1 + 0.4% AcOH, *v*/*v*/*v*): R_f = 0.41. **^{1}H-NMR** (300 MHz, [D$_6$]DMSO, 308 K): δ = 1.37 (s, 9 H, $C(CH_3)_3$), 1.22–1.86 (m, 6 H, γ-H$_2$, δ-H$_2$, ε-H$_2$), 2.22–2.37 (m, 2 H, α-H$_2$), 3.50 (t, ^3J = 6.7 Hz, 2 H, ζ-H$_2$), 3.63–3.78 (m, 1 H, β-H), 6.62 (d, ^3J = 8.9 Hz, 1 H, NH-Boc) ppm. **^{13}C-NMR** (125 MHz, [D$_6$]DMSO, 308 K): δ = 28.18 (C($\underline{C}H_3)_3$), 24.03, 31.89, 33.29 (γ-CH$_2$, δ-CH$_2$, ε-CH$_2$), 34.89 (ζ-CH$_2$), 38.99–39.99 (α-CH$_2$ covered from [D$_6$]DMSO signal), 47.02 (β-CH), 77.35 ($\underline{C}(CH_3)_3$), 154.98 (Boc-$\underline{C}$O), 172.69 (COOH) ppm. **MS** (ESI): *m/z* = 346.1 [*M* + Na]$^+$, 362.1 [*M* + K]$^+$, 322.1 [*M*–- H]$^-$. **HR-MS** (ESI): $C_{12}H_{22}BrNO_4$ calculated 346.0624 [*M* + Na]$^+$, 362.0364 [*M* + K]$^+$, 322.0659 [*M* – H]$^-$, found 346.0629 [*M* + Na]$^+$, 362.0364 [*M* + K]$^+$, 322.0661 [*M* – H]$^-$.

[1] The moderate yield of 50% for the hydroxy-substituted product **7** was caused by classical competition between substitution and elimination reactions. The formation of the elimination product (23%) under the required conditions (pH = 9.5, 65 °C.) was unavoidable, which is why the reaction was monitored by thin layer chromatography (TLC) and stopped immediately after the starting material had completely converted.
[2] The waste product of triphyenylphospine, triphenylphospine oxide, was difficult to completely separate under applied conditions.

(9) (*R*)-3-((*tert*-Butoxycarbonyl)amino)-7-(2,4,6-trioxo-1,3,5-triazinan-1-yl)heptanoic acid, Boc-D-β³-hLys(CYA)-OH

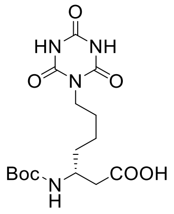

$C_{15}H_{24}N_4O_7$ [372.38]

Under inert gas atmosphere, (*R*)-7-bromo-3-((*tert*-butoxycarbonyl)amino) heptanoic acid (**8**) (605 mg, 1.87 mmol, 1.00 eq) and cyanuric acid (1.20 g, 9.33 mmol, 5.00 eq) were suspended in dry DMF (12 mL) followed by dropwise addition of 2,3,4,6,7,8,9,10-octahydropyrimido[1,2-*a*]azepine (DBU) (279 µL, 285 mg, 1.87 mmol, 1.00 eq). The reaction mixture was heated to 70 °C and stirred for 2 d. The solvent was removed under reduced pressure and the residue was suspended in H_2O (50 mL). Then, the pH was adjusted to 5 using an aqueous 1 M HCl solution and DCM (50 mL) was added. The white precipitate, cyanuric acid, was filtered off and the layers were separated. The aqueous phase was extracted with DCM (2 × 50 mL). The combined organic phases were dried over $MgSO_4$ and adsorbed on silica gel. The crude product was purified via column chromatography using the eluents DCM/MeOH (96:4 + 0.5% AcOH, *v*/*v*/*v*) → DCM/MeOH (9:1 + 0.5% AcOH, *v*/*v*/*v*). AcOH was removed as an azeotropic mixture with toluene (3 x 20 mL) to give the product **9** (605 mg, 1.87 mmol, 52%) as a white solid. **TLC** (DCM/MeOH, 9:1 + 0.5% AcOH, *v/v/v*): R_f = 0.35. **^{1}H-NMR** (300 MHz, [D6]DMSO, 308 K): δ = 1.36 (s, 9 H, $C(CH_3)_3$), 1.14–1.56 (m, 6 H, γ-H_2, δ-H_2, ε-H_2), 2.15–2.36 (m, 2 H, α-H_2), 3.54–3.75 (m, 3 H, β-H, ζ-H_2), 6.56–6.65 (m, 1 H, NH-Boc), 11.39 (s_{br}, 1 H, COOH) ppm. **^{13}C-NMR** (125 MHz, [D6]DMSO, 308 K): δ = 28.21 (C($\underline{C}H_3)_3$), 22.73, 27.26, 34.00 (γ-CH_2, δ-CH_2, ε-CH_2), 38.99−40.23 (α-CH_2 covered from [D6]DMSO signal), 40.36 (ζ-CH_2), 47.32 (β-CH), 77.34 ($\underline{C}(CH_3)_3$), 148.72, 149.86, 149.52 (3 × CYA-CO), 154.98 (Boc-$\underline{C}$O), 173.29 (COOH) ppm. **MS** (ESI): *m/z* = 395.2 [*M* + Na]$^+$, 411.1 [*M* + K]$^+$, 371.2 [*M* – H]$^-$, 743.3 [2*M* – H]$^-$. **HR-MS** (ESI): $C_{15}H_{24}N_4O_7$ calculated 395.1537 [*M* + Na]$^+$, 371.1572 [*M* – H]$^-$, found 395.1536 [*M* + Na]$^+$, 371.1574 [*M* – H]$^-$.

(10) (*R*)-3-((((9*H*-Fluoren-9-yl)methoxy)carbonyl)amino)-7-(2,4,6-trioxo-1,3,5-triazinan-1-yl)heptanoic acid, Fmoc-D-β³-hLys(CYA)-OH

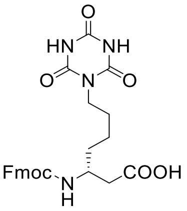

$C_{25}H_{26}N_4O_7$ [494.50]

Boc-D-β³-hLys(CYA)-OH (**9**) (163 mg, 438 µmol, 1.00 eq) was cooled to 0 °C and dissolved with TFA /H_2O (95:5, 7 mL, *v/v*). After stirring 1 h at 0 °C and 1 h at RT, TFA was removed under a gentle stream of nitrogen. The residual was coevaporated with toluene (3 × 10 mL) and Et_2O (10 mL) dried *in vacuo* and lyophilized. The Boc-deprotected intermediate was then dissolved in H_2O (3 mL) and $NaHCO_3$ (56.7 mg, 675 µmol, 1.54 eq) and cooled to 0 °C. A solution of Fmoc-OSu (228 mg, 676 µmol, 1.54 eq) in dioxane (3 mL) was added dropwise, and the mixture was stirred first 1 h at 0 °C and then overnight at RT. After adding H_2O (20 mL), the pH was adjusted to 1 with an aqueous 1 M HCl solution. The aqueous layer was extracted with DCM (4 × 30 mL) and the combined extracts were dried over $MgSO_4$ and adsorbed on silica gel. The crude product was purified by column chromatography using the eluents DCM/MeOH (96:4 + 0.5% AcOH, *v/v/v*) → DCM/MeOH (9:1 + 0.5% AcOH, *v/v/v*) and AcOH was removed as an azeotropic mixture with toluene (3 x 20 mL). The pure product **10** (195 mg, 394 µmol, 90%) was isolated as a white solid. **TLC** (DCM/MeOH, 9:1 + 0.5% AcOH, *v/v/v*): R_f = 0.43. **^{1}H-NMR** (300 MHz, [D6]DMSO, 308 K): δ = 1.14–1.61 (m, 6 H, γ-H_2, δ-H_2, ε-H_2), 2.15–2.37 (m, 2 H, α-H_2), 3.61 (t, ^3J = 7.3 Hz, 2 H, ζ-H_2), 3.69–3.80 (m, 1 H, β-H), 4.15–4.36 (m, 3 H, Fmoc-CH, Fmoc-CH_2), 7.18 (d, ^3J = 7.8 Hz, 1 H, NH-Fmoc), 7.30–7.43 (m, 4 H,

4 × Fmoc-CH_{Ar}), 7.68 (d, ^3J = 7.3 Hz, 2 H, 2 × Fmoc-CH_{Ar}), 7.87 (d, ^3J = 7.4 Hz, 2 H, 2 × Fmoc-CH_{Ar}) ppm. **^{13}C-NMR** (125 MHz, [D$_6$]DMSO, 308 K): δ = 22.68, 27.25, 33.87 (γ-CH_2, δ-CH_2, ε-CH_2), 38.50–39.99 (α-CH_2 covered from [D$_6$]DMSO signal), 40.31 (ζ-CH_2), 46.78 (Fmoc-CH), 47.69 (β-CH), 65.06 (Fmoc-CH_2), 119.96, 125.13, 126.95, 127.48, 128.83 (8 × Fmoc-CH_{Ar}), 140.65, 143.80, 143.92 (4 × Fmoc-C_{Ar}), 148.72, 149.86 (3 × CYA-CO), 155.45 (Fmoc-<u>C</u>O), 173.24 (COOH) ppm. **MS** (ESI): *m/z* = 517.2 [*M* + Na]$^+$, 533.2 [*M* + K]$^+$, 493.2 [*M* – H]$^-$, 987.4 [2*M* – H]$^-$. **HR-MS** (ESI): $C_{25}H_{26}N_4O_7$ calculated 517.1694 [*M* + Na]$^+$, 533.1433 [*M* + K]$^+$, 493.1729 [*M* – H]$^-$, found 517.1688 [*M* + Na]$^+$, 533.1422 [*M* + K]$^+$, 493.1729 [*M* – H]$^-$.

(11) (R)-3-((*tert*-Butoxycarbonyl)amino)-7-((4,6-diamino-1,3,5-triazin-2-yl)amino)heptanoic acid, Boc-D-β^3-hLys(TAT)-OH

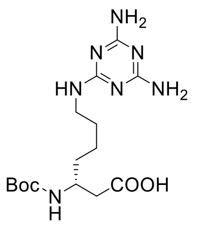

$C_{15}H_{27}N_7O_4$ [369.43]

Boc-D-β^3-hLys-OH (**10**) (3.00 g, 11.5 mmol, 1.00 eq), K_2CO_3 (3.18 g, 23.0 mmol, 2.00 eq) and 2,4-diamino-6-chlorotriazine (3.35 g, 23.0 mmol, 2.00 eq) were suspended in DMSO (55 mL) and stirred at 90 °C for 18.5 h. DMSO was removed under reduced pressure and MeOH (50 mL) was added. The white precipitate, excess 6-chloro-2,4-diamino-1,3,5-triazine, was filtered off and washed with MeOH (3 × 10 mL). The solvent was removed under reduced pressure and the residue was lyophilized. The crude product was resuspended in MeOH (50 mL), adsorbed on silica gel and purified by column chromatography using the eluents acetone/H_2O (20:1 → 10:1, *v/v*). The product **11** (2.99 g, 8.09 mmol, 70%) was obtained as a white solid. **TLC** (Aceton/H_2O, 10:1, *v/v*): R_f = 0.39. **^{1}H-NMR** (300 MHz, [D$_6$]DMSO, 308 K): δ = 1.36 (s, 9 H, C(CH_3)$_3$), 1.15–1.46 (m, 6 H, γ-H_2, δ-H_2, ε-H_2), 1.99–2.14 (m, 2 H, α-H_2), 3.10–3.17 (m, 2 H, ζ-H_2), 3.50–3.63 (m, 1 H, β-H), 6.05 (s_{br}, 4 H, 2 × NH_2), 6.43 (t, ^3J = 5.6 Hz, 1 H, TAT-NH), 6.93 (d, ^3J = 8.7 Hz, 1 H, NH-Boc) ppm. **^{13}C-NMR** (125 MHz, [D$_6$]DMSO, 308 K): δ = 28.29 (C(<u>C</u>H_3)$_3$), 23.38, 29.34, 34.24 (γ-CH_2, δ-CH_2, ε-CH_2), 38.50–39.99 (α-CH_2 covered from [D$_6$]DMSO signal), 42.17 (ζ-CH_2), 48.02 (β-CH), 76.94 (<u>C</u>(CH_3)$_3$), 154.70 (Boc-<u>C</u>O), 166.06, 166.85 (3 × TAT-C), 175.67 (COOH) ppm. **MS** (ESI): *m/z* = 370.2 [*M* + H]$^+$, 392.2 [*M* + Na]$^+$, 368.2 [*M* – H]$^-$. **HR-MS** (ESI): $C_{15}H_{27}N_7O_4$ calculated 370.2197 [*M* + H]$^+$, 368.2052 [*M* – H]$^-$, found 370.2195 [*M* + H]$^+$, 368.2052 [*M* – H]$^-$.

(12) (*R*)-3-((((9*H*-Fluoren-9-yl)methoxy)carbonyl)amino)-7-((4,6-diamino-1,3,5-triazin-2-yl)amino)heptanoic acid, Fmoc-D-β^3-hLys(TAT)-OH

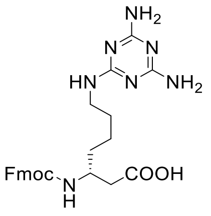

$C_{25}H_{29}N_7O_4$ [491.55]

Boc-D-β^3-hLys(TAT)-OH (**11**) (1.01 g, 2.73 mmol, 1.00 eq) was dissolved in TFA/H_2O (95:5, 10 mL, *v/v*) at 0 °C and stirred first 1 h at 0 °C followed by 1 h at RT. Then, TFA was removed under gentle stream of N_2 and the residue was coevaporated with toluene (3 × 15 mL) and Et_2O (2 × 15 mL), dried *in vacuo* and lyophilized. The D-β^3-hLys(TAT)-OH·TFA was then dissolved in H_2O (10 mL) and neutralized with a saturated $NaHCO_3$-solution. Additional $NaHCO_3$ (344 mg, 4.01 mmol, 1.50 eq) was added and the mixture was cooled to 0 °C. Fmoc-OSu (1.38 g, 4.01 mmol, 1.50 eq) in dioxane (10 mL) was added dropwise to the solution and the mixture was stirred first 1 h at 0 °C and then overnight at RT. After adding H_2O (10 mL), the mixture was extracted with Et_2O (3 × 10 mL). The combined organic layers were discarded. The aqueous phase was adjusted to a pH of 1 with an aqueous 1 M HCl solution. The precipitate was filtered off, washed with H_2O/MeOH (3 × 15 mL) and dried *in vacuo*. The compound **12** (861 mg, 1.75 mmol, 64%) was obtained as a white solid. **^{1}H-NMR** (300 MHz, [D$_6$]DMSO, 308 K): δ = 1.11–1.54 (m, 6 H, γ-H_2, δ-H_2, ε-H_2), 2.16–2.43 (m, 2 H, α-H_2),

3.04–3.24 (m, 2 H, ζ-H_2), 3.71–3.85 (m, 1 H, β-H), 4.21–4.30 (m, 3 H, Fmoc-CH, Fmoc-CH_2), 5.99 (s_{br}, 4 H, 2 × NH_2), 6.39 (s_{br}, 1 H, TAT-NH), 7.17 (d, ^3J = 8.1 Hz, 1 H, NH-Fmoc), 7.32–7.43 (m, 4 H, 4 × Fmoc-CH_{Ar}), 7.67–7.69 (m, 2 H, 2 × Fmoc-CH_{Ar}), 7.86–7.88 (m, 2 H, 2 × Fmoc-CH_{Ar}) ppm. **^{13}C-NMR** (125 MHz, [D6]DMSO, 308 K): *δ* = 22.68, 25.17, 29.15, 33.90 (γ-CH_2, δ-CH_2, ε-CH_2), 38.50–39.99 (α-CH_2, ζ-CH_2 covered from [D6]DMSO signal), 46.76 (Fmoc-CH), 47.94 (β-CH), 65.08 (Fmoc-CH_2), 119.98, 125.08, 126.95, 127.51, 128.83 (8 × Fmoc-CH_{Ar}), 140.65, 143.77, 143.89 (4 × Fmoc-C_{Ar}), 155.47 (Fmoc-CO), 166.21, 166.74, 166.99 (3 × TAT-C), 172.62 (COOH) ppm. **MS** (ESI): *m/z* = 492.2 $[M + H]^+$, 490.2 $[M - H]^-$. **HR-MS** (ESI): $C_{25}H_{29}N_7O_4$ calculated 492.2354 $[M + H]^+$, 490.2208 $[M – H]^-$, found 492.2354 $[M + H]^+$, 490.2209 $[M – H]^-$.

3. 3. β^3-D-Peptides

3.1 Synthetic Scheme

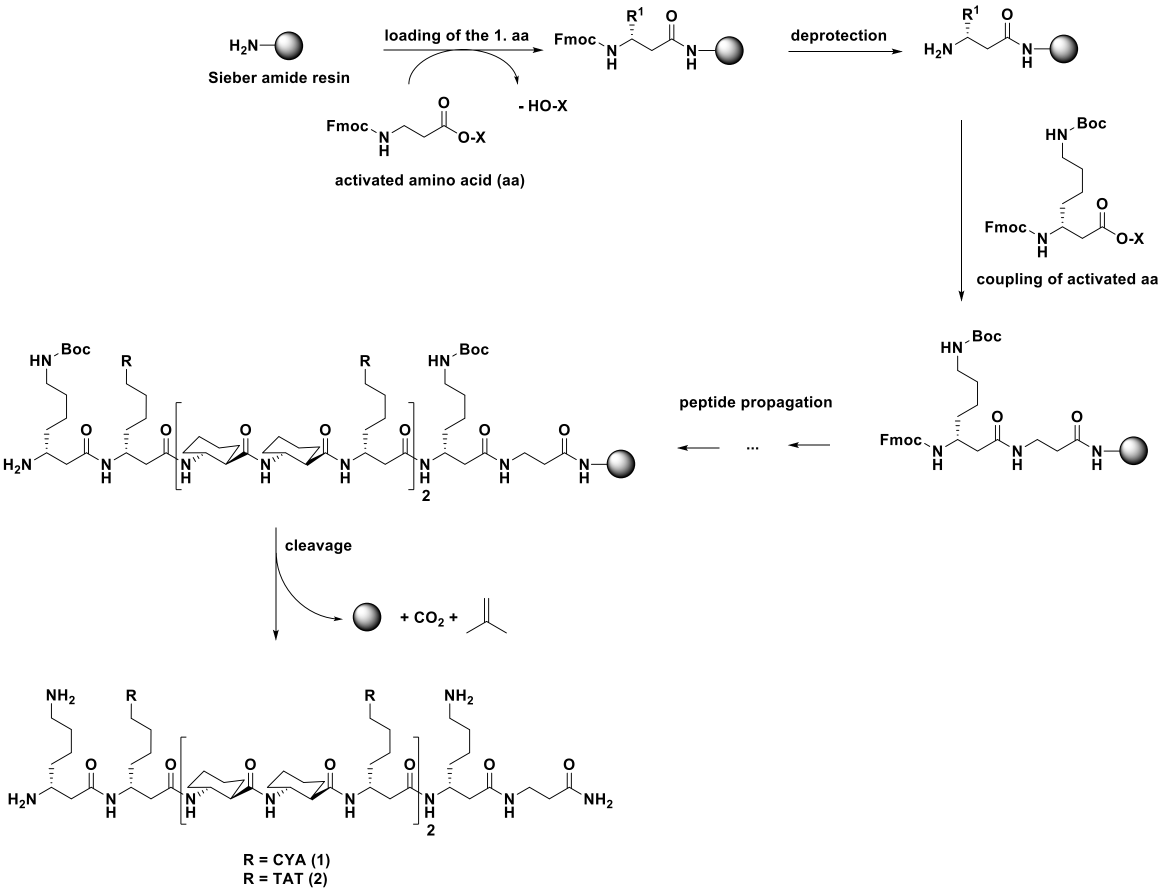

Scheme S2. Schematic representation of Fmoc-based SPPS with orthogonally-protected β^3-D-amino acids. The peptide synthesis proceeds from the *C*- to the *N*-terminus. The abbreviations are defined as follows: Boc = *tert*-butyloxycarbonyl, Fmoc = fluorenylmethoxycarbonyl, and X = leaving group.

3.2 General Synthetic Procedure

GSP 3: Loading of the first amino acid on resin

As solid support Sieber amide resin (0.76 mmol/g) was applied for all syntheses. The resin was transferred to a DiscarditTMII-syringe (Becton Dickinson, BD, Fraga, Spain) with a polyethylen-frit. The resin was swollen in dichloromethane (DCM)/*N*,*N*-dimethylformamide (DMF) (1:1, *v*/*v*) for 1 h prior to any coupling reaction. Afterwards, the resin was treated with 20% piperidine

in *N*-methyl-2-pyrrolidone (NMP) or DMF under microwave irradiation (30 s, 50 W, 50 °C and 180 s, 50 W, 50 °C) followed by successive washing with NMP, DCM, NMP or DMF, DCM, DMF (3 × each). For the coupling, an activated solution consisting of Fmoc-β-Ala-OH (first coupling: 5.0 eq/ second coupling: 3.0 eq), 1-[bis(dimethylamino)methylene]-1*H*-1,2,3-triazolo[4,5-b]pyridinium 3-oxide hexafluorophosphate (HATU, 4.5 eq/2.7 eq) and 3-hydroxytriazolo[4,5-b]pyridine (HOAt, 5.0 eq/3.0 eq) and *N*-ethyl-*N*-(propan-2-yl)propan-2-amine (DIEA, 10 eq) in DMF (40 µL/µmol) was added to the resin followed by microwave irradiation (10 min, 25 W, 50 °C). Double-coupling was performed. After successive wash with NMP or DMF, DCM, methanol, DCM (3 × each), the resin was stored under vacuum. The loading density was estimated via UV analysis of the Fmoc-deprotection product dibenzofulvene following the procedure described by GUDE.[5]

GSP 4: Microwave-assisted manual Fmoc-based SPPS

Fmoc-based SPPS consisted of a cycle with the steps in the following order: Fmoc-deprotection (1), washing (2), first amino acid coupling (3), washing (2), second amino acid coupling (3), washing (2). The deprotection and coupling reactions were microwave-assisted. The batch size for each experiment was stated correspondingly. After transfer of the resin to a DiscarditTMII-syringe with PE-frit, a solution of dichloromethane (DCM)/*N*,*N*-dimethylformamide (DMF) (1:1, *v*/*v*) was added followed by swelling at RT for 1 h.

(1) **Deprotection** of the Fmoc-group was achieved by applying 20% piperidine in DMF or NMP (*v*/*v*, 2 ×, 30 sec, 50 W, 50 °C and 3 min, 50 °C, 50 W).

(2) **Washing** of the resin in between all steps was performed in the order DMF, NMP, DCM, DMF (3 ×) or NMP, DMF, DCM, NMP (3 ×).

(3) **Coupling** was realized by adding of an activated solution consisting of the amino acid (5.0 eq/3.0 eq), HOAt (5.0 eq/3.0 eq), HATU (4.5 eq/2.7 eq) and DIEA (10 eq) in DMF (450 µL per 10 µM resin) to the resin followed by microwave irradiation (25 W, 60 °C, 10 min). The coupling of Fmoc-ACHC-OH (**9**) was adapted from a procedure described by GELLMANN[6]: an activated solution of the amino acid (5.0 eq/3.0 eq), HATU (4.5 eq/2.7 eq), HOAt (5.0 eq/3.0 eq) and DIEA (10 eq) in 0.8 M LiCl[3]/NMP (40 µL/µmol) was given to the resin followed by microwave-irradiation (10 min, 25 W, 45 °C). The coupling time for a second ACHC right after an already coupled ACHC was expanded to 20 min. For each amino acid a double-coupling was performed. The cycle was repeated until complete sequence was achieved. After final deprotection, the resin was washed with NMP, DMF, DCM, NMP, DCM, MeOH, DCM (3×, respectively), dried under vacuum and stored at –21 °C until been further processed.

GSP 5: Peptide cleavage from solid support

For the cleavage, a concentrated solution of trifluoroacetic acid (TFA)/*m*-Cresol (95:5, *v*/*v*) was prepared and diluted to 10% in DCM. The diluted solution was transferred to the resin from GSP 4. The solution was filtered off after 1–2 h shaking at RT and the resin was washed twice with the diluted cleavage solution. The combined filtrates were mixed with the concentrated solution and incubated for 0.5–1 h at room temperature to reach full deprotection of protecting groups (here Boc). After cleavage, excess of TFA was removed by a gentle N_2 stream. The product was precipitated in –21 °C cold Et_2O (3 × 8 mL) and centrifuged for 30 min at 9000 rpm and –10 to 0 °C. The supernatant was discarded. The procedure of precipitation and centrifugation was repeated twice and the crude product was dried in vacuum. The crude peptide was dissolved in aqueous CH_3CN and purified by HPLC followed by freeze-drying of the purified product and storage at –21 °C.

3.3 Synthesis and Characterization

(1) H-D-β^3-hLys-D-β^3-hLys(CYA)-*R*,*R*-ACHC-*R*,*R*-ACHC-D-β^3-hLys(CYA)-*R*,*R*-ACHC-*R*,*R*-ACHC-D-β^3-hLys(CYA)-D-β^3-hLys-β-Ala-NH_2

---

[3] Chaotropic salts were used to prevent sequence-length-dependent aggregations on the resin and thereby to increase the coupling efficiency.

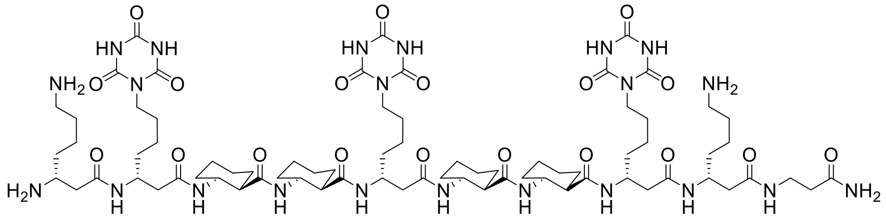

$C_{75}H_{122}N_{22}O_{19}$ [1635.94]

The peptide **1** (15.0 µmol) was prepared according to GSP 4 by a manual SPPS on a Sieber amide resin (0.74 mmol/g) pre-loaded with Fmoc-β-Ala (GSP 3). The amino acids Fmoc-D-β^3-hLys(CYA)-OH (**10**), Fmoc-D-β^3-hLys(Boc)-OH (**13**) and Fmoc-*R*,*R*-ACHC-OH (**14**) were used for the synthesis. Then the peptide **1** (7.50 µmol) was cleaved from the solid support according to GSP 5. After HPLC purification (semi-preparative, gradient: 25 → 45% B in 30 min), the pure peptide **1** was obtained as a white solid. **HPLC** (analytical, gradient: 25 → 45% B in 30 min): t_R = 21.40 min. **MS** (ESI): *m/z* = 546.0 [*M* + 3H]$^{3+}$, 818.5 [*M* + 2H]$^{2+}$, 1636.0 [*M* + H]$^+$. **HR-MS** (ESI): $C_{75}H_{122}N_{22}O_{19}$ calculated 818.4701 [*M* + 2H]$^{2+}$, 1635.9329 [*M* + H]$^+$, found 818.4706 [*M* + 2H]$^{2+}$, 1635.9327 [*M* + H]$^+$.

(2) H-D-β^3-hLys-D-β^3-hLys(TAT)-*R*,*R*-ACHC-*R*,*R*-ACHC-D-β^3-hLys(TAT)-*R*,*R*-ACHC-*R*,*R*-ACHC-D-β^3-hLys(TAT)-D-β^3-hLys-β-Ala-NH$_2$

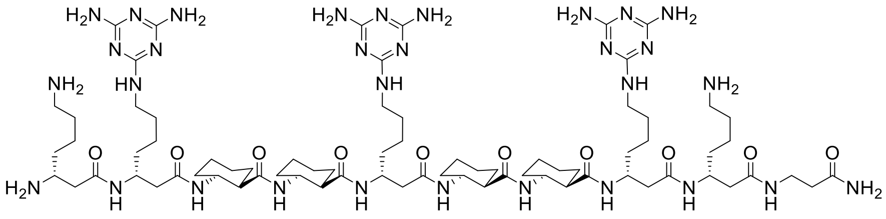

$C_{75}H_{131}N_{31}O_{10}$ [1627.08]

The peptide **2** (20.0 µmol) was prepared according to GSP 4 by a manual SPPS on a Sieber amide resin (0.74 mmol/g) pre-loaded with Fmoc-β-Ala (GSP 3). The amino acids Fmoc-D-β^3-hLys(TAT)-OH (**12**), Fmoc-D-β^3-hLys(Boc)-OH (**13**) and Fmoc-*R*,*R*-ACHC-OH (**14**) were used for the sequence. Subsequently, the peptide **2** (10.0 µmol) was cleaved from the solid support according to GSP 5. After HPLC purification (semi-preparative, gradient: 25 → 40% B in 30 min), the peptide **2** was obtained as a white solid. **HPLC** (analytical, gradient: 25 → 40% B in 30 min): t_R = 19.75 min. **MS** (ESI): *m/z* = 407.8 [*M* + 4H]$^{4+}$, 543.0 [*M* + 3H]$^{3+}$, 814.1 [*M* + 2H]$^{2+}$, 1627.1 [*M* + H]$^+$. **HR-MS** (ESI): $C_{75}H_{131}N_{31}O_{10}$ calculated 543.0304 [*M* + 3H]$^{3+}$, 814.0420 [*M* + 2H]$^{2+}$, 1627.0768 [*M* + H]$^+$, found 543.0299 [*M* + 3H]$^{3+}$, 814.0419 [*M* + 2H]$^{2+}$, 1627.0781 [*M* + H]$^+$.

4. Aggregation Studies

Peptide stock solutions for each peptide (1 mM) were prepared and stored at –21 °C until usage. Prior aggregation experiments, buffer stock solution consist of triethylammonium acetate buffer (TEAA) (10 mM, pH = 7.3–7.4) or ammonium acetate (10 mM, pH = 7.0–7.1) was prepared. For the experiment, peptide stock solutions were warmed up to RT and ultrasonicated for 10 min, cooled down to RT and centrifuged for 1 min at maximum speed with a standard lab desk centrifuge. The samples were prepared as stated in the corresponding sub section below.

4.1 Circular Dichroism (CD)- and Ultraviolet (UV) Spectroscopy

CD spectra were measured on a JASCO J-810A spectropolarimeter (Groß-Umstadt, Germany) equipped with a *JASCO* PTC-423S peltier control unit. The device was purged with nitrogen before and during the operations. All experiments were carried out in a 1.0 mm

quartz glass cuvette of HELLMA (Suprasil® QS) and the temperature was controlled by the sensor in the holder. The following parameters were set the experiments: *Data Mode* CD and Absorption, *Band Width* 1.0 nm, *Response* 2 s, *Sensitivity* 100 mdeg, *Datapitch* 0.2 nm, *Scanning speed* 100 nm/min, *Accumulations* 5. The spectra were background-corrected against solvent and cuvette length (1 cm) and smoothed with SAVITZKY-GOLAY filter. The data was plotted as molar ellipticity θ_{molar} (deg $cm^2 dmol^{-1}$) vs wavelength (nm) following the equation $\Theta_{molar} = 100 \times \frac{CD}{c \cdot l}$. *CD* represents measured ellipticity in deg, *c* = final sample concentration [mol cm^{-3}] and *l* = cuvette length [cm].

Temperature-dependent CD measurements:

β^3-CYA decapeptide **1** and β^3-TAT decapeptide **2** were diluted each in aqueous TEAA buffer (5 mM, pH = 7.3) to a final concentration of 20 µM per peptide. The temperature was enhanced after each measurement in steps of 20 beginning with 0 °C to 80 °C. Temperature-dependent CD spectra were also recorded for the equimolar mixture **1** and **2** (20 µM in 5 mM in TEAA buffer, pH = 7.4, at 20 °C). For these purpose, a total peptide concentration of 50 µM in 5 mM TEAA buffer was prepared, incubated overnight at 4 °C, diluted prior measurement to 20 µM and recorded at different temperatures, starting at 0 °C to 80 °C.

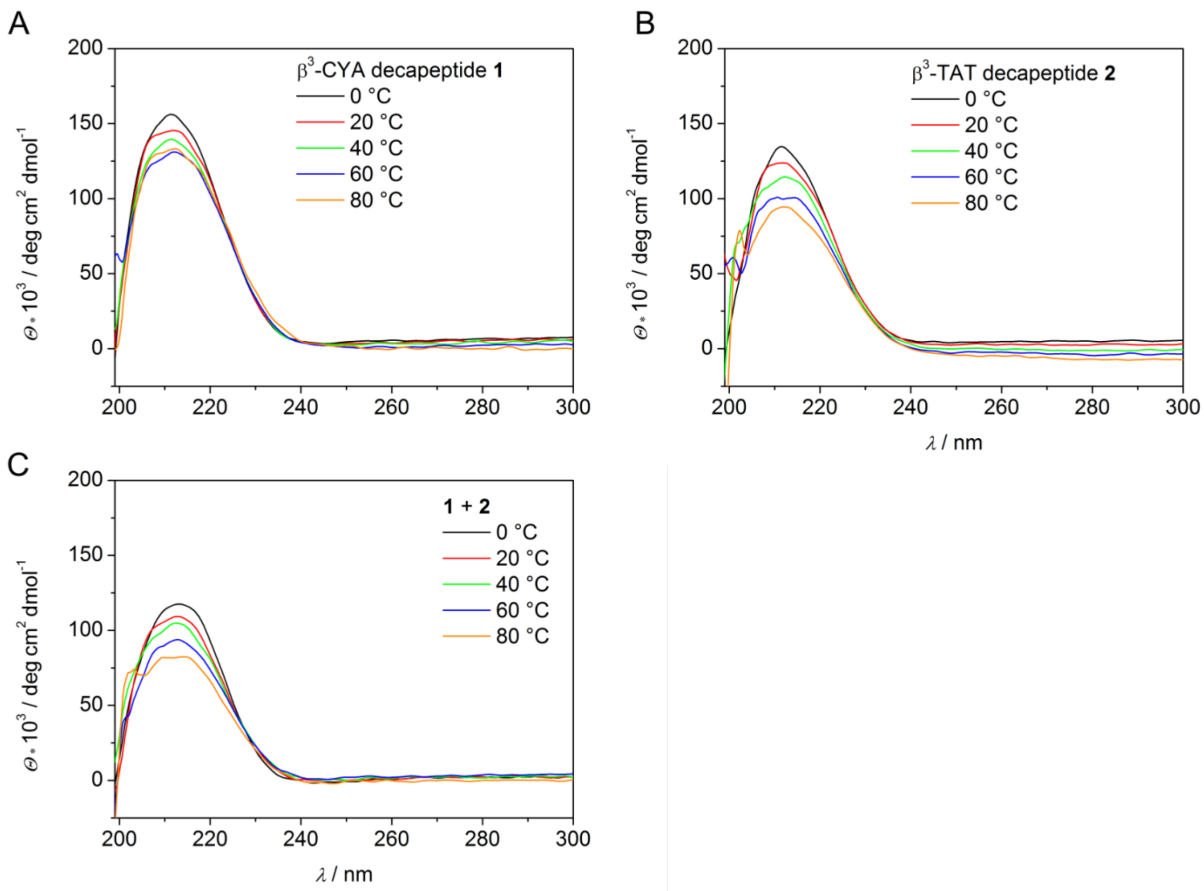

Figure S1. Temperature-dependent CD-spectra of **A** β^3-CYA decapeptide **1** (20 µM in 5 mM TEAA buffer, pH = 7.3) (maximum at 211 nm), **B** β^3-TAT decapeptide **2** (20 µM in 5 mM TEAA buffer, pH = 7.3) (maximum at 211 nm) and **C** an equimolar mixture of peptides **1** and **2** (50 µM diluted to 20 µM in 5 mM TEAA buffer, pH = 7.3) (maximum at 212 nm).

Concentration- and time-dependent CD measurements:

CD spectra were performed from equimolar mixture of **1** and **2**. Therefore, peptides **1** and **2** were mixed at different concentrations ranging from 50 to 500 µM, measured within 20 min and after overnight incubation at 4 °C. After CD measurements, UV spectrum of each sample was recorded for concentration- and time-dependent UV measurement (see Figure S3 C and D).

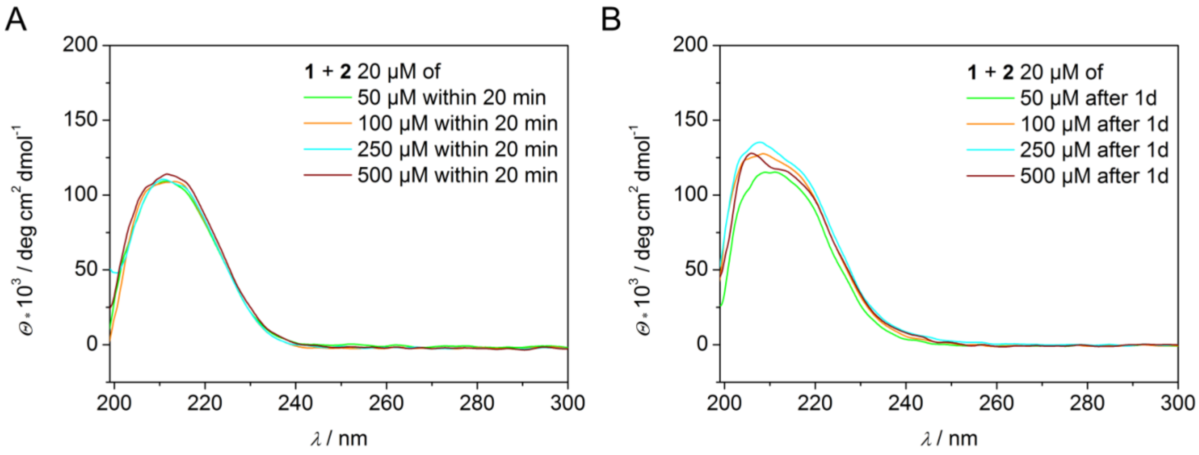

Figure S2. Concentration- and time-dependent CD spectra of **A** equimolar mixture of β3-CYA decapeptide **1** and β3-TAT decapeptide **2** (in 5 mM TEAA buffer, pH = 7.3) measured within 20 min (maxima at 211 nm), **B** equimolar mixture of **1** and **2** (in 5 mM TEAA buffer, pH = 7.3) after 1 d incubation (maximum shift from 211→206 nm).

UV spectra were measured on a JASCO V-550 UV/vis spectropolarimeter (Groß-Umstadt, Germany). All experiments were carried out in a 1.0 mm quartz glass cuvette of HELLMA (Suprasil® QS). The following parameters were set the experiments: *Photometric Mode* Absorption, *Band Width* 0.5 or 1.0 nm, *Response* Quick or Fast, *Datapitch* 0.5 or 1.0 nm, *Scanning speed* 400 nm/min, *Accumulations* 5. The spectra were background-corrected against solvent and cuvette length (1 cm) and smoothed with SAVITZKY-GOLAY filter. The data was plotted as Absorbance (AU) vs. wavelength (nm).

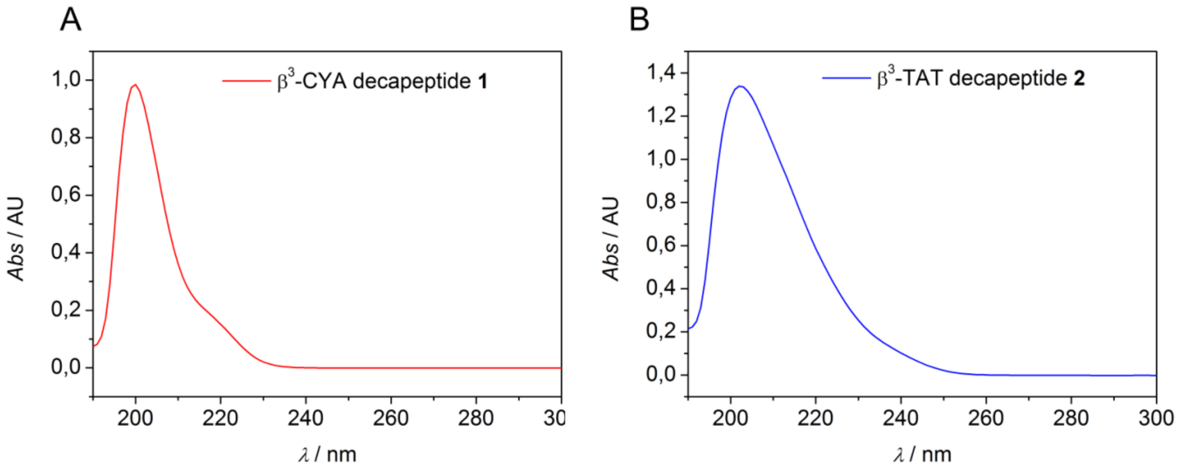

Figure S3. UV spectra of **A** β3-CYA decapeptide **1** (20 µM in 5 mM TEAA buffer, pH = 7.3) (maximum at 200 nm, shoulder at 219 nm) and **B** β3-TAT decapeptide **2** (20 µM in 5 mM TEAA buffer, pH = 7.3) (maximum at 202 nm).[4]

[4] The maximum at 200 nm correspond to β3-peptides, while the shoulder at 219 nm in **A** belongs to CYA. The absorption of CYA is pH-dependent leading in neutral form to no or a weak band, while at pH 7.4 due to pKa1 6.5 partially protonated.[7,8] TAT with first pKa 5.1 is neutral at pH 7.4. In this form it has a weak band at 235 nm, caused by symmetry-forbidden π-electron-transitions.[7–9]

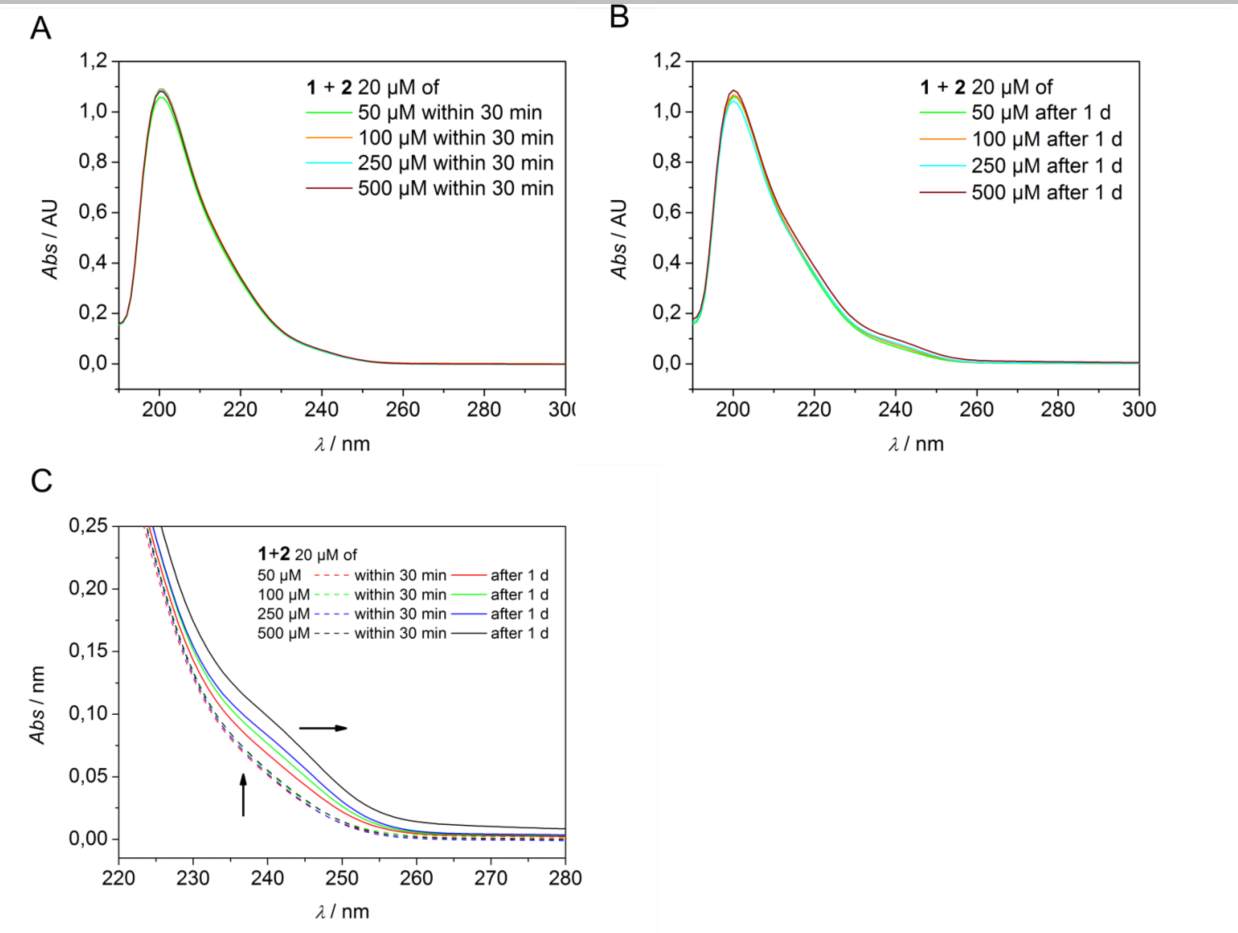

Figure S4. Concentration- and time-dependent UV spectra of **A** equimolar mixture of β³-CYA decapeptide **1** and β³-TAT decapeptide **2** (in 5 mM TEAA buffer, pH = 7.3) within 30 min incubation (maximum at 201 nm) and **B** equimolar mixture of **1** and **2** (in 5 mM TEAA buffer, pH = 7.3) after 1 d incubation (maximum 200 nm). **C** Plot of A and B together followed by amplification of the region corresponding to the weak band in B. In contrast to the results within 30 min, a slight red-shift and an increase in absorbance were obtained after one day incubation (indicated with arrows).

4.2 Dynamic Light Scattering (DLS)

DLS measurements of the individual peptides and of the equimolar mixture were performed on a Zetasizer Nano S from MALVERN PANALYTICAL (Malvern, United Kingdom) with a laser (633 nm) using 12 mm disposal polystyrene (PS) cuvettes. Therefore, solutions of single peptides (125 µM in 5 mM TEAA buffer, pH = 7.4) and their equimolar mixtures (125 or 500 µM in 5 mM TEAA buffer, pH = 7.4) were prepared, vortexed (10 s), centrifuged (10 s) and passed through a 0.2 µm filter[5] to the PS cuvette directly. Afterwards, the samples were incubated overnight at 4 °C. All measurements were recorded with a detection angle of 173° at 20 °C. The intensity distribution resulted from several measurements (15 runs per 200 s). The temperature (20 °C), the viscosity of the solvent (η_{H2O} = 1.0031 mPa·s) and the refractive index for Proteins (n_D^{20} = 1.450) were taken into account to get the volume and number diameter curves.

[5] DLS measurements are very sensitive to typical dust particles present in the environment of the sample.[10] This impurities scatter light more strongly than the nano-sized molecules. Therefore, filtration of the sample before mixing was crucial. Ultracentrifugation after filtration was not performed here.

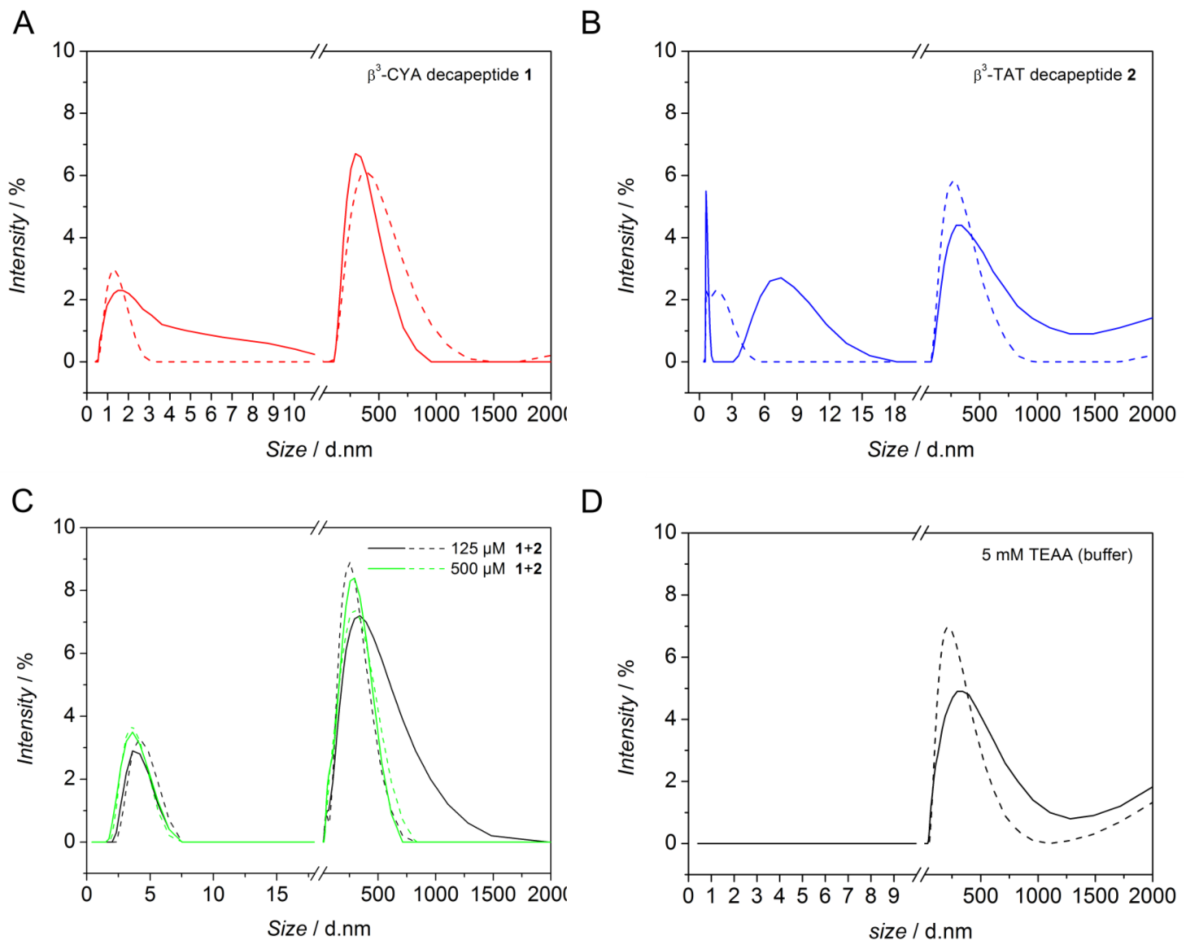

Figure S5. DLS intensity size distribution of **A** β^3-CYA decapeptide **1** (125 µM in 5 mM TEAA buffer, pH = 7.4), **B** β^3-TAT decapeptide **2** (125 µM in 5 mM TEAA buffer, pH = 7.4), **C** an equimolar mixture of peptides **1** and **2** (125 µM or 500 µM in 5 mM TEAA) and **D** 5 mM TEAA buffer with measurement 1 (solid line) and 2 (dashed line), respectively. The higher hydrodynamic diameter sizes observed after 30 nm belong to the buffer TEAA (see D). Broad distributions are observed when the monomers were measured individually, indicating non-specific homomeric interactions in A and B. The equimolar mixtures **1** and **2** show broad peak as a consistent trend than observed for the single peptides in C.

4.3 Electrospray Ionization (ESI) Mass Spectrometry

The data shown for the aggregates were recorded on a micrOTOF-Q II instrument from BRUKER DALTONIK (Bremen, Germany). An equimolar mixture of β^3-CYA decapeptide **1** (25 µM) and β^3-TAT decapeptide **2** (25 µM) in 5 mM ammonium acetate (pH = 7.1) was prepared, vortexed (10 s), centrifuged (10 s) and incubated overnight at 4 °C. Prior measurement, the sample was diluted to a final concentration of 20 µM (10 µM each peptide) and injected with a flow rate of 8 µL min^{-1}. The ESI source in the positive mode was operated at an ESI voltage of –4500 V for capillary and -500 V for end plate offset with nitrogen as nebulizer gas (5 L min^{-1} flow rate) and drying gas (0.7 bar backing pressure, 80 °C temperature).

Theoretical mass ratios were calculated using envipat isotope pattern calculator from EAWAG (Dübendorf, Switzerland).[11]

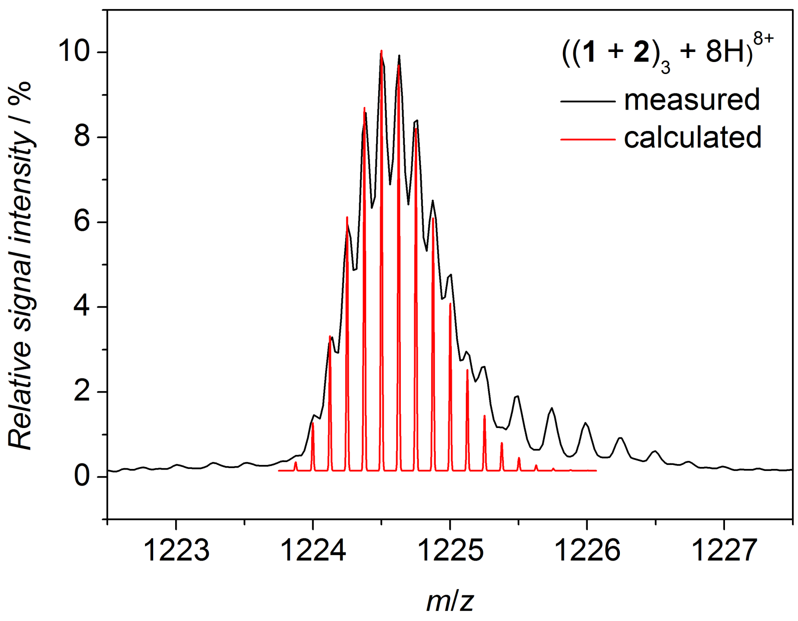

Figure S6. Measured (black) and calculated (red) isotopic pattern of ((**1** + **2**)$_3$ + 8H)$^{8+}$ from positive mode ESI-MS spectrum.

Table S1. Sum formulas, measured and theoretical *m*/*z* ratios of the most abundant ions* obtained from ESI-MS measurement in 5 mM ammonium acetate.

Assignment	Sum formula	Measured	Theoretical
(**1** + 3H)$^{3+}$	$C_{75}H_{125}N_{22}O_{19}{}^{3+}$	545.981	545.983
(**2** + 3H)$^{3+}$	$C_{75}H_{134}N_{31}O_{10}{}^{3+}$	543.029	543.031
(**1** + 2H)$^{2+}$	$C_{75}H_{124}N_{22}O_{19}{}^{2+}$	818.466	818.471
(**2** + 2H)$^{2+}$	$C_{75}H_{133}N_{31}O_{10}{}^{2+}$	814.041	814.043
(**1** + **2** + 3H)$^{3+}$	$C_{150}H_{256}N_{53}O_{29}{}^{3+}$	1088.341	1088.341
((**1** + **2**)$_3$ + 7H)$^{7+}$	$C_{450}H_{766}N_{159}O_{87}{}^{7+}$	1399.291	1399.294
((**1** + **2**)$_3$ + 8H)$^{8+}$	$C_{450}H_{767}N_{159}O_{87}{}^{8+}$	1224.506	1224.508

*signals above 0.5% relative intensity.

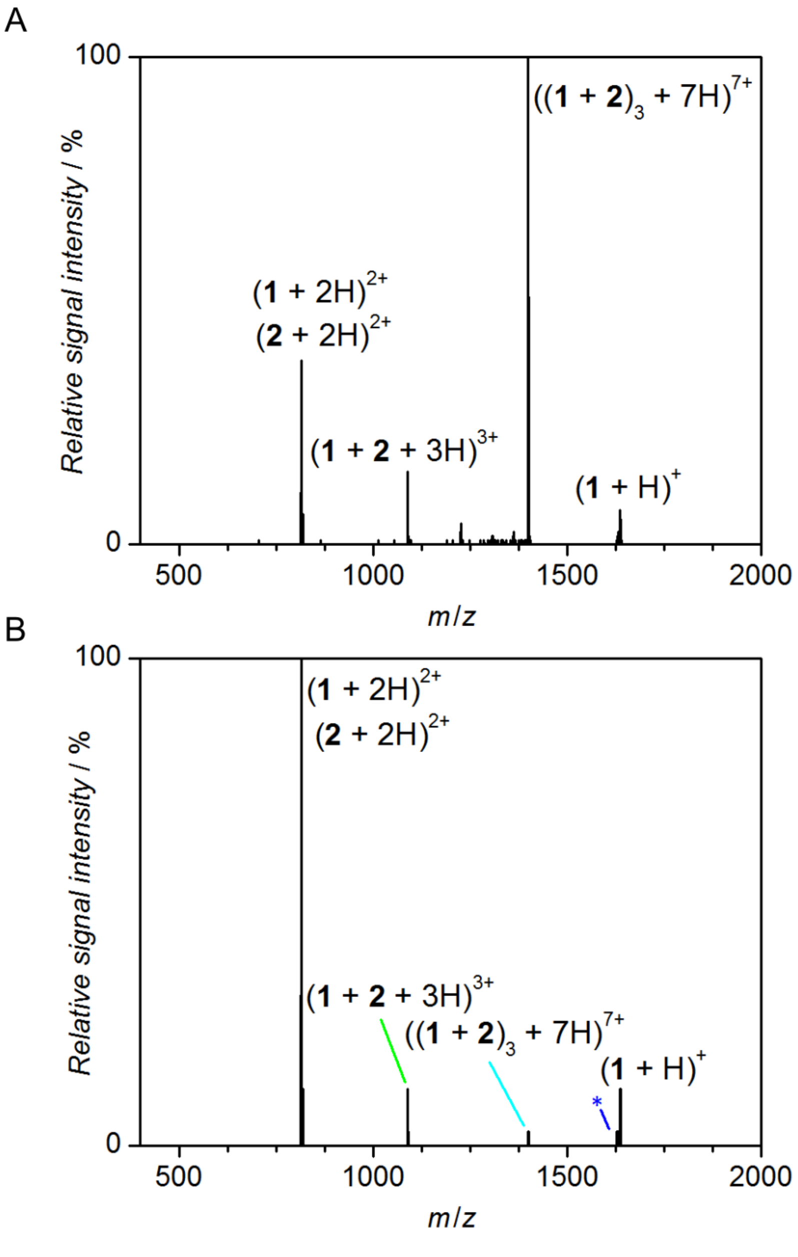

Figure S7. Collision-induced dissociation of selected mass. A ESI-MS spectrum of depicted mass ((**1** + **2**)$_3$ + 7H)$^{7+}$ at 0 V and **B** its fragment ions enhanced by higher voltage (7 V). *determined as (**2** + H)$^+$.

5. Supporting Figures and Spectra

5.1 ^{1}H- and ^{13}C-NMR-Spectra of β^3-D-Amino Acids

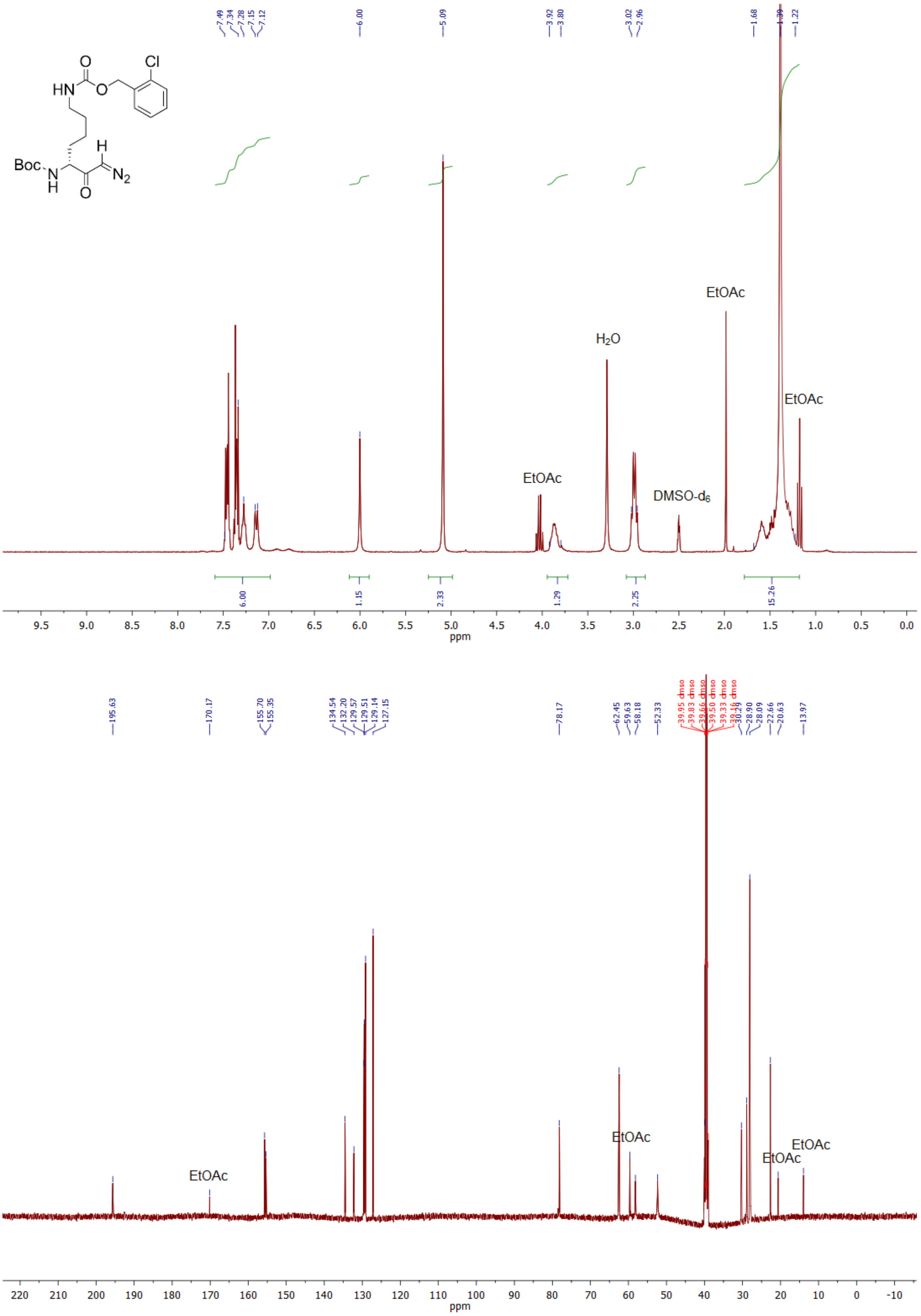

Figure S8. ^{1}H- (above) and ^{13}C-NMR (below) of (*R*)-3-((*tert*-butoxycarbonyl)-amino)-7-((((2-chlorobenzyl)-oxy)-carbonyl)-amino)-1-diazoheptane-2-on, Boc-D-Lys(2-Cl-Cbz)-CHN_2 (**4b**).

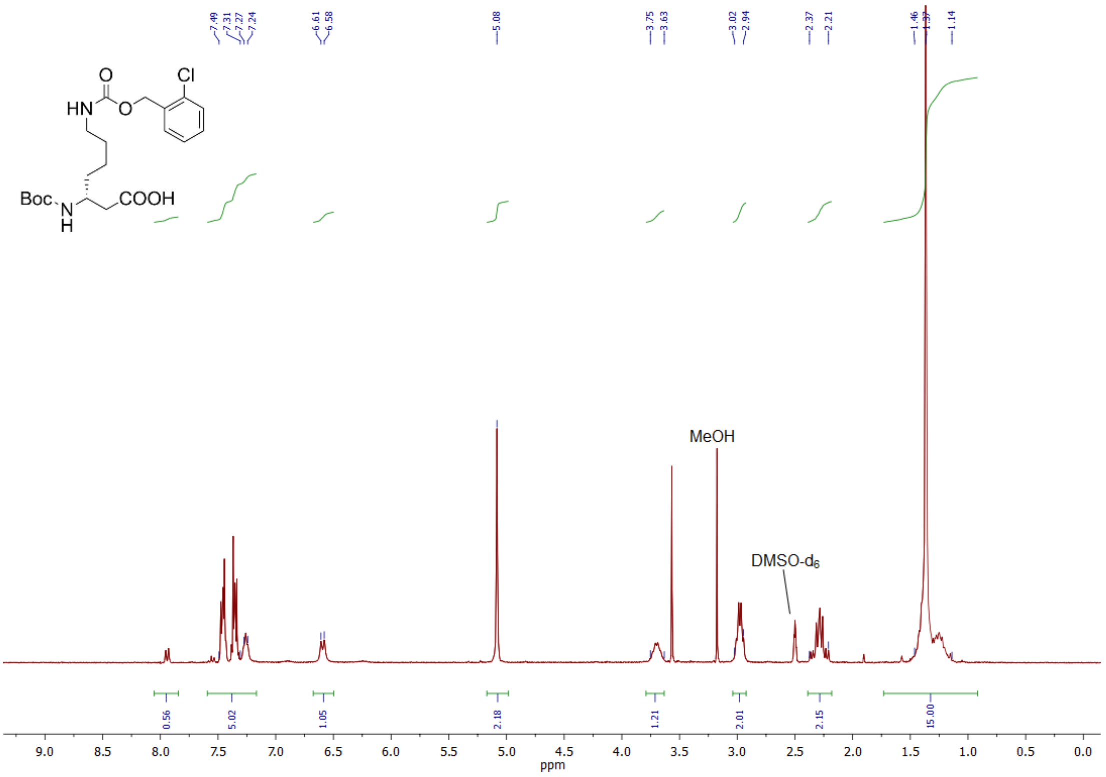

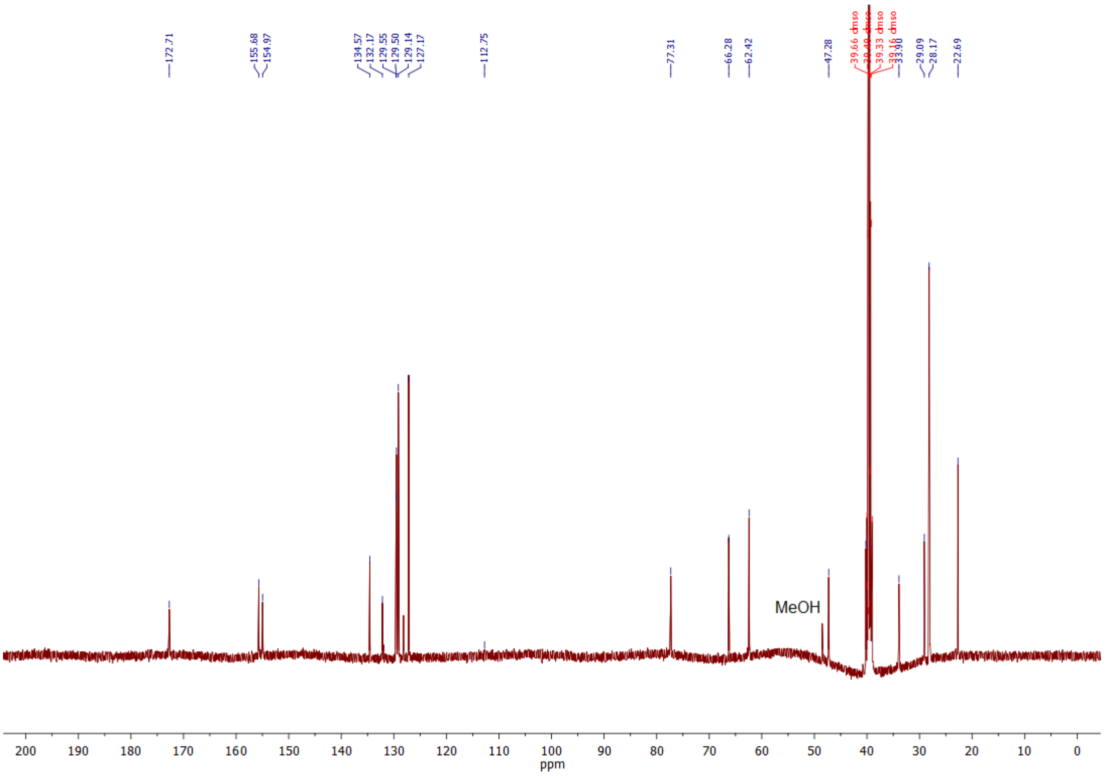

Figure S9. ^{1}H- (above) and ^{13}C-NMR (below) of (*R*)-3-((*tert*-butoxycarbonyl)amino)-7-((((2-chlorobenzyl)oxy)carbonyl)-amino)heptanoic acid, Boc-D-β^{3}-hLys(2-Cl-Cbz)-OH (**5b**).

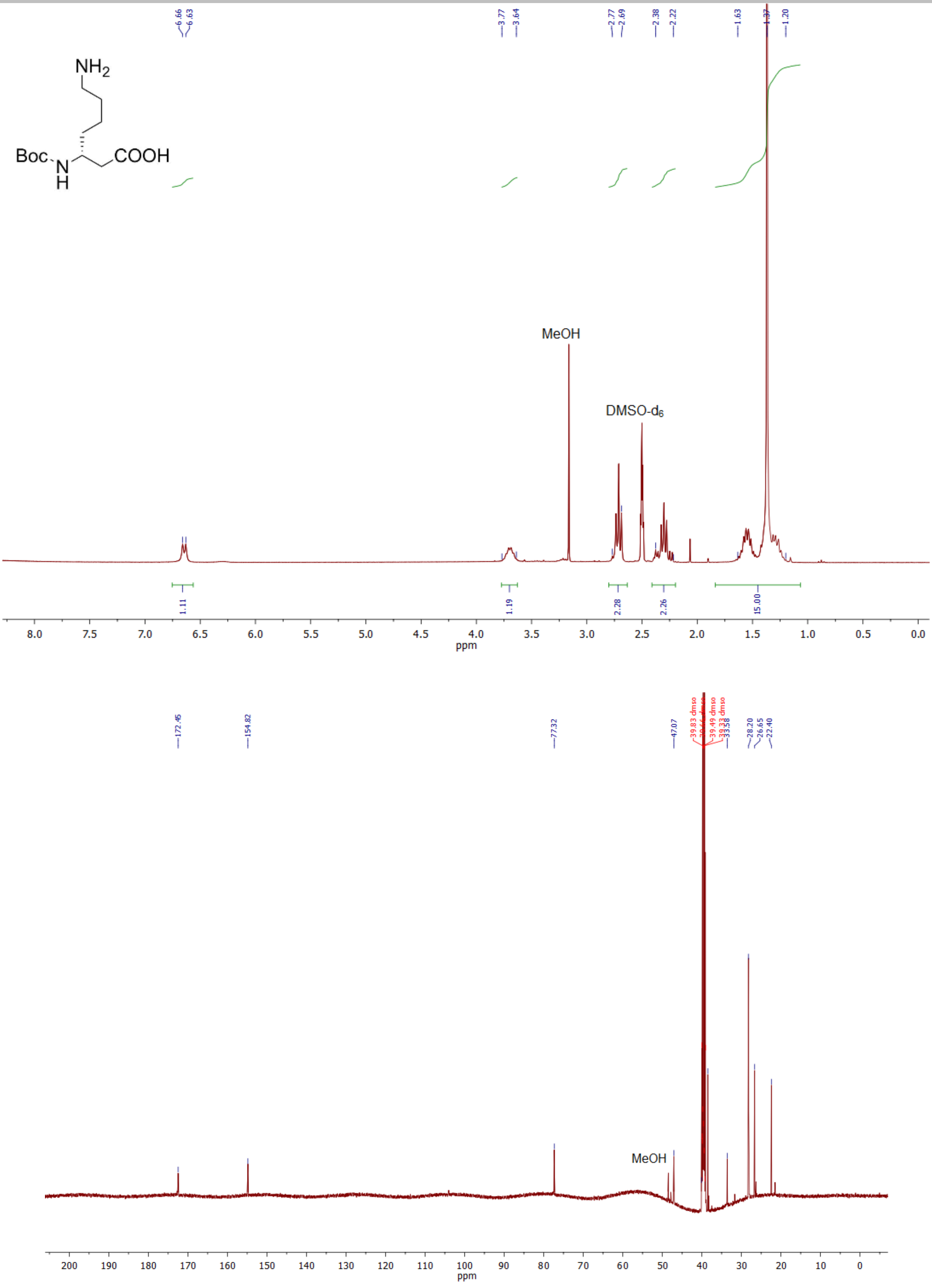

Figure S10. ^{1}H- (above) and ^{13}C-NMR (below) of (*R*)-7-amino-3-((*tert*-butoxycarbonyl)amino)heptanoic acid, Boc-D-β^{3}-hLys-OH (**6**).

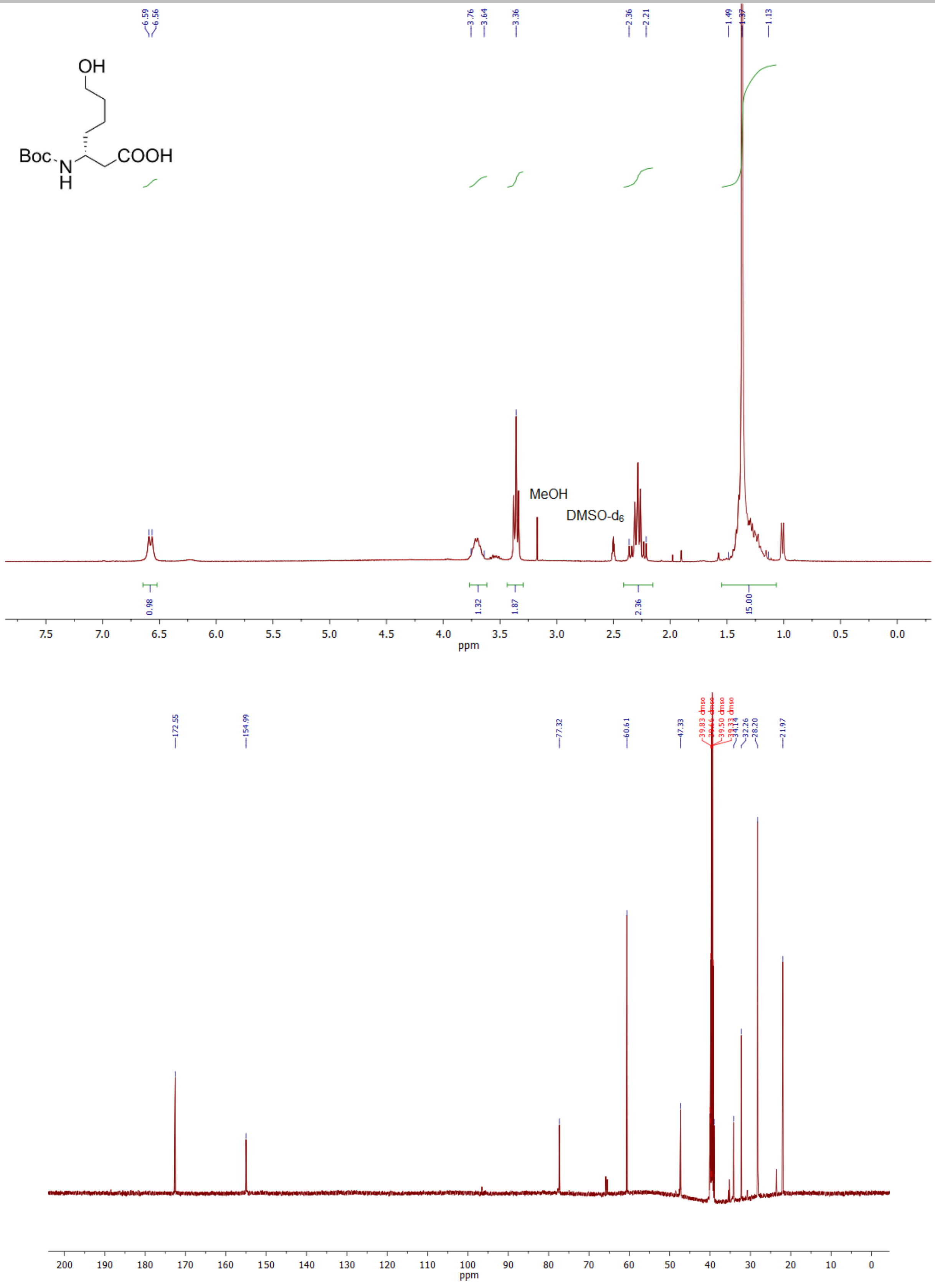

Figure S11. ^{1}H- (above) and ^{13}C-NMR (below) of (*R*)-3-((*tert*-butoxycarbonyl)amino)-7-hydroxyheptanoic acid (**7**).

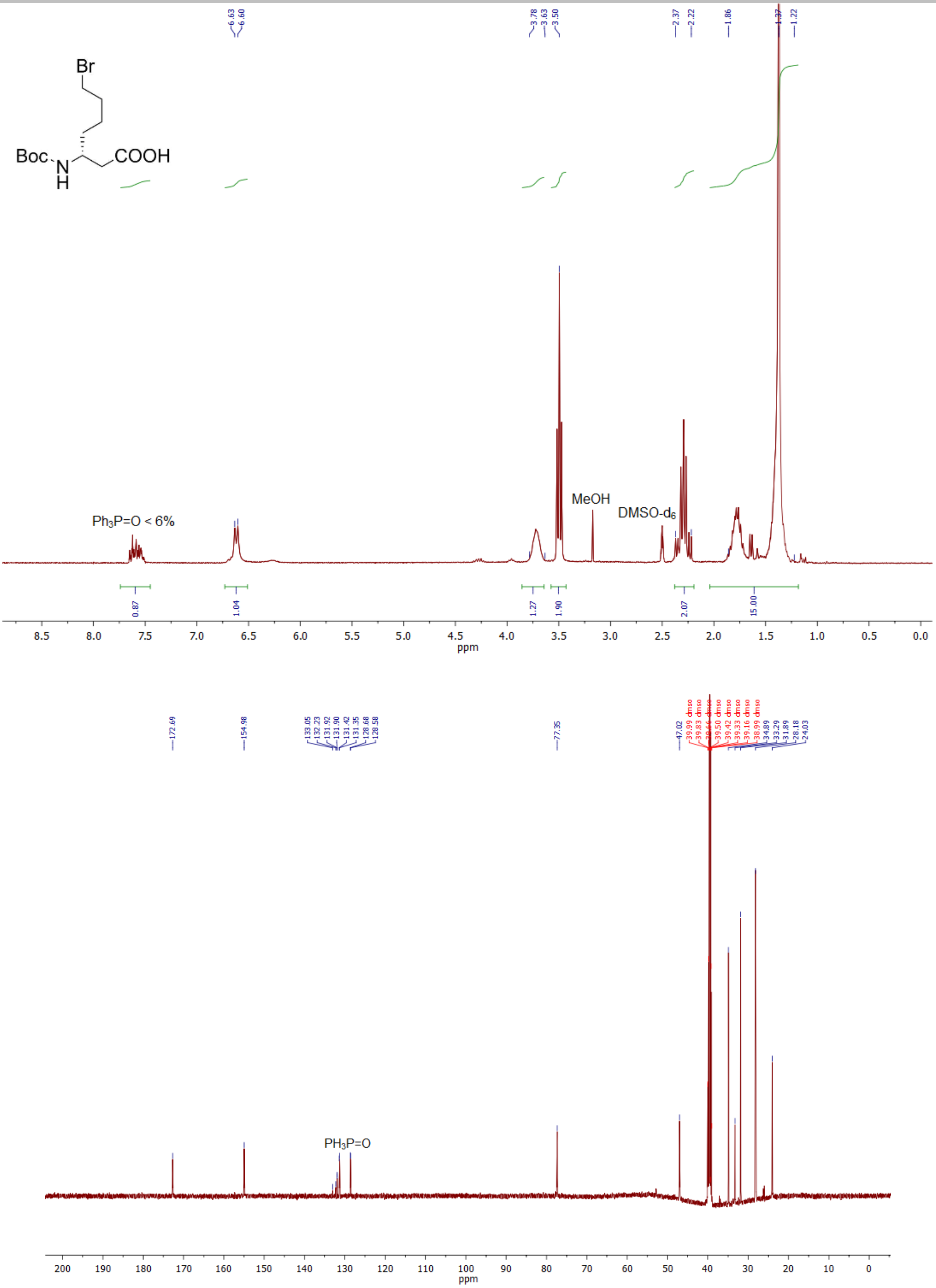

Figure S12. ^{1}H- (above) and ^{13}C-NMR (below) of (*R*)-7-bromo-3-((*tert*-butoxycarbonyl)amino)heptanoic acid (**8**).

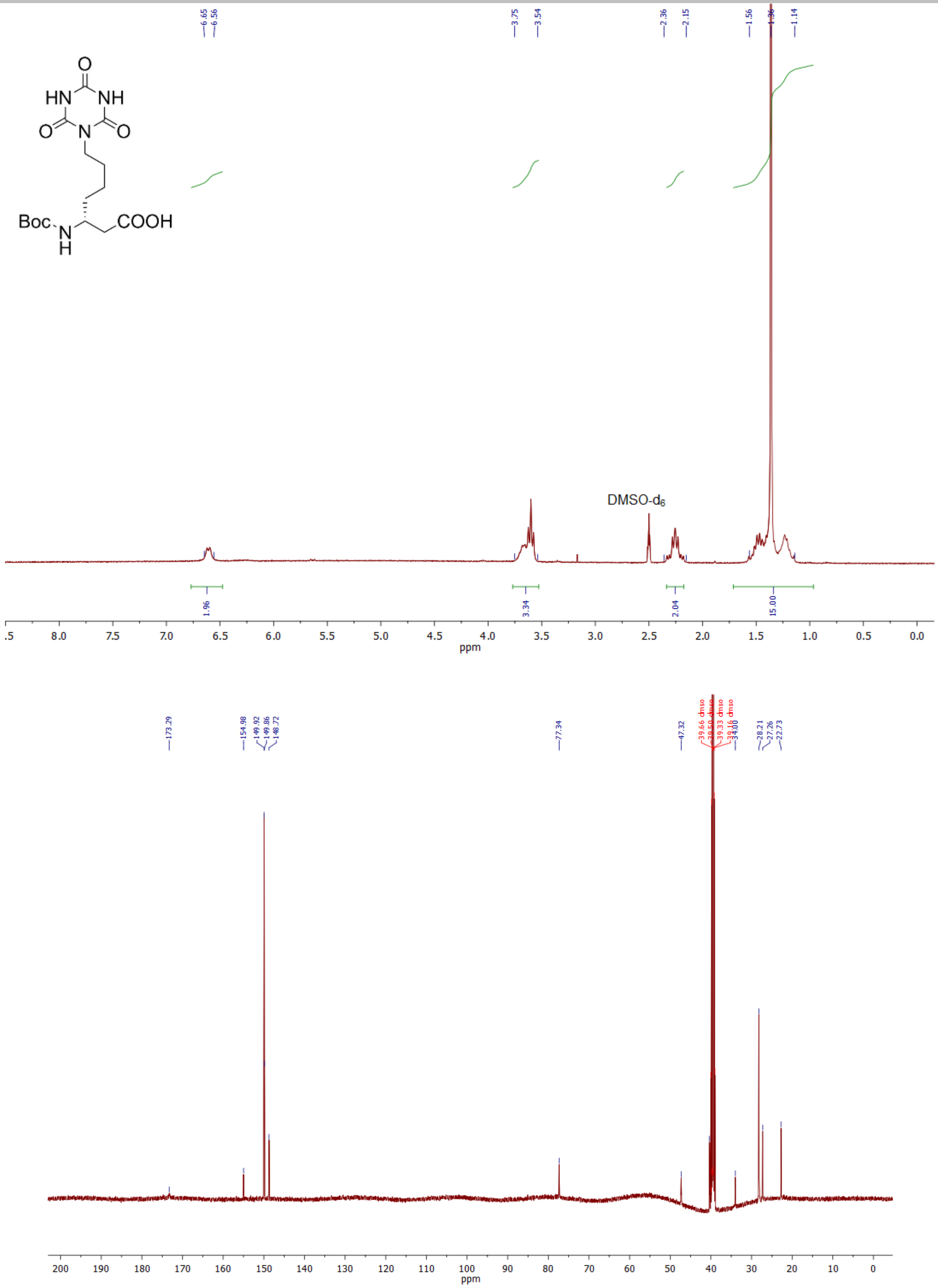

Figure S13. ^{1}H- (above) and ^{13}C-NMR (below) of (*R*)-3-((*tert*-butoxycarbonyl)amino)-7-(2,4,6-trioxo-1,3,5-triazinan-1-yl)heptanoic acid, Boc-D-β^3-hLys(CYA)-OH (**9**).

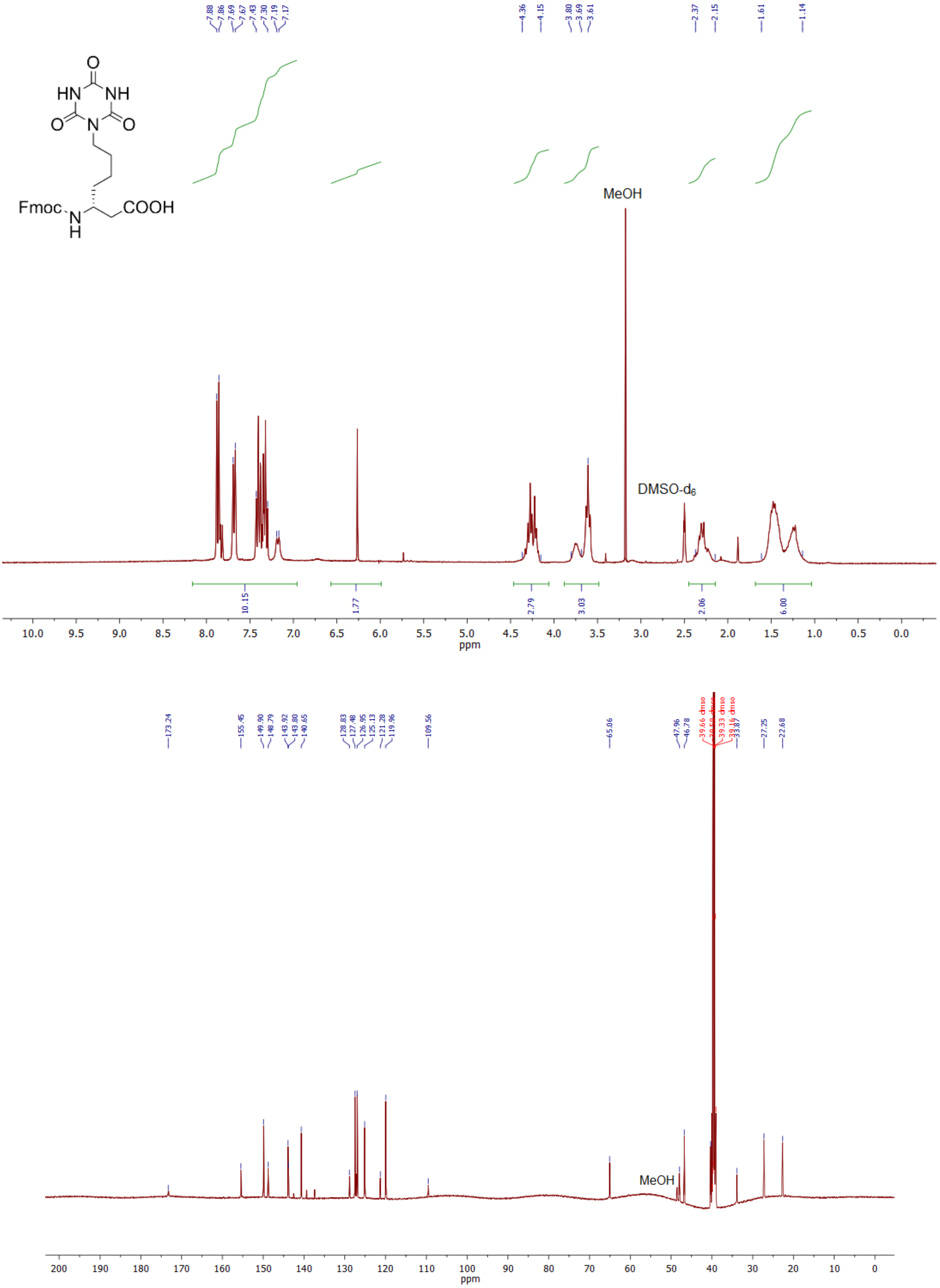

Figure S14. ^{1}H- (above) and ^{13}C-NMR (below) of (*R*)-3-((((9*H*-fluoren-9-yl)methoxy)carbonyl)amino)-7-(2,4,6-trioxo-1,3,5-triazinan-1-yl)heptanoic acid, Fmoc-D-β^3-hLys(CYA)-OH (**10**).

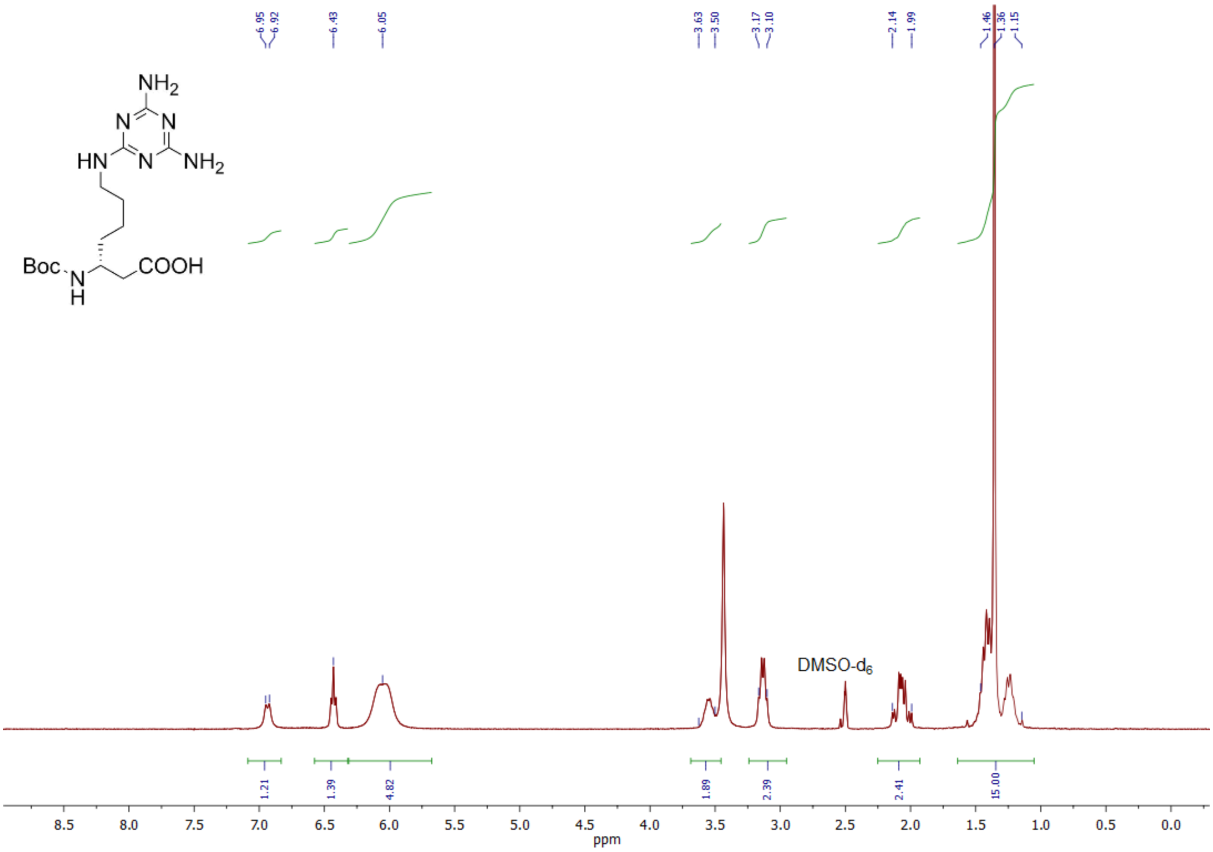
NH2
N N
HN N NH2
Boc N COOH
H
DMSO-d6
ppm

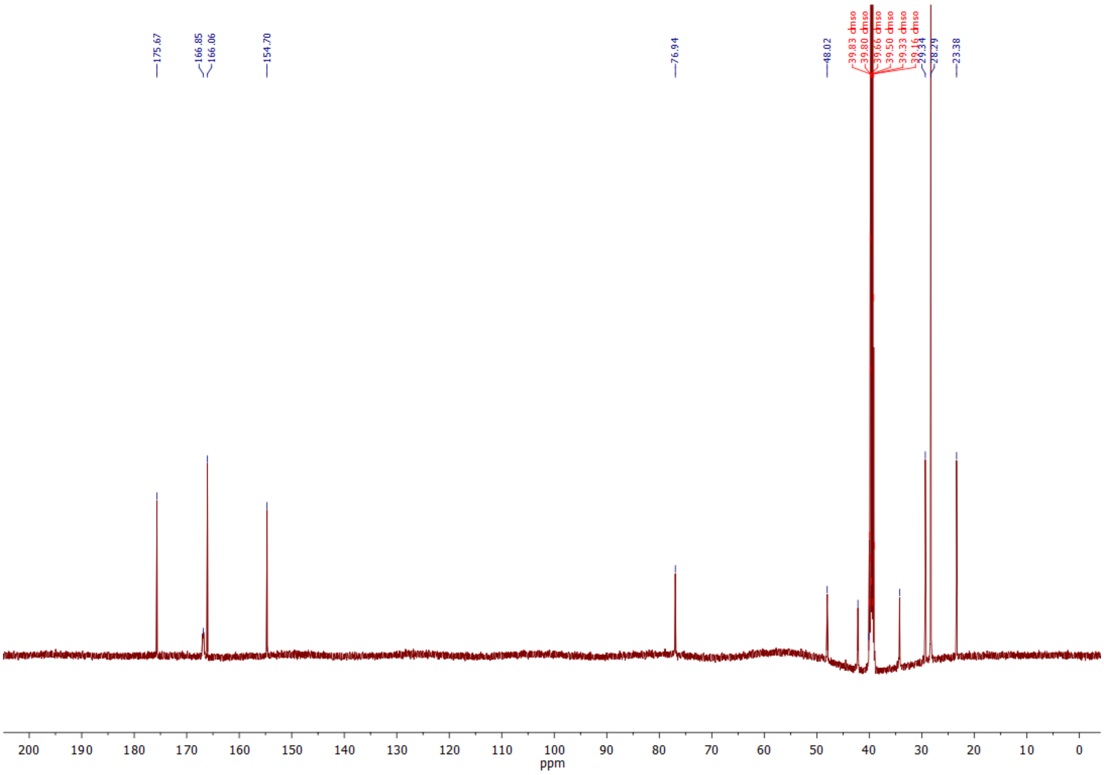
ppm

Figure S15. ^{1}H- (above) and ^{13}C-NMR (below) of (*R*)-3-((((9*H*-fluoren-9-yl)methoxy)carbonyl)amino)-7-(2,4,6-trioxo-1,3,5-triazinan-1-yl)heptanoic acid, Fmoc-D-β[3]-hLys(CYA)-OH (**11**).

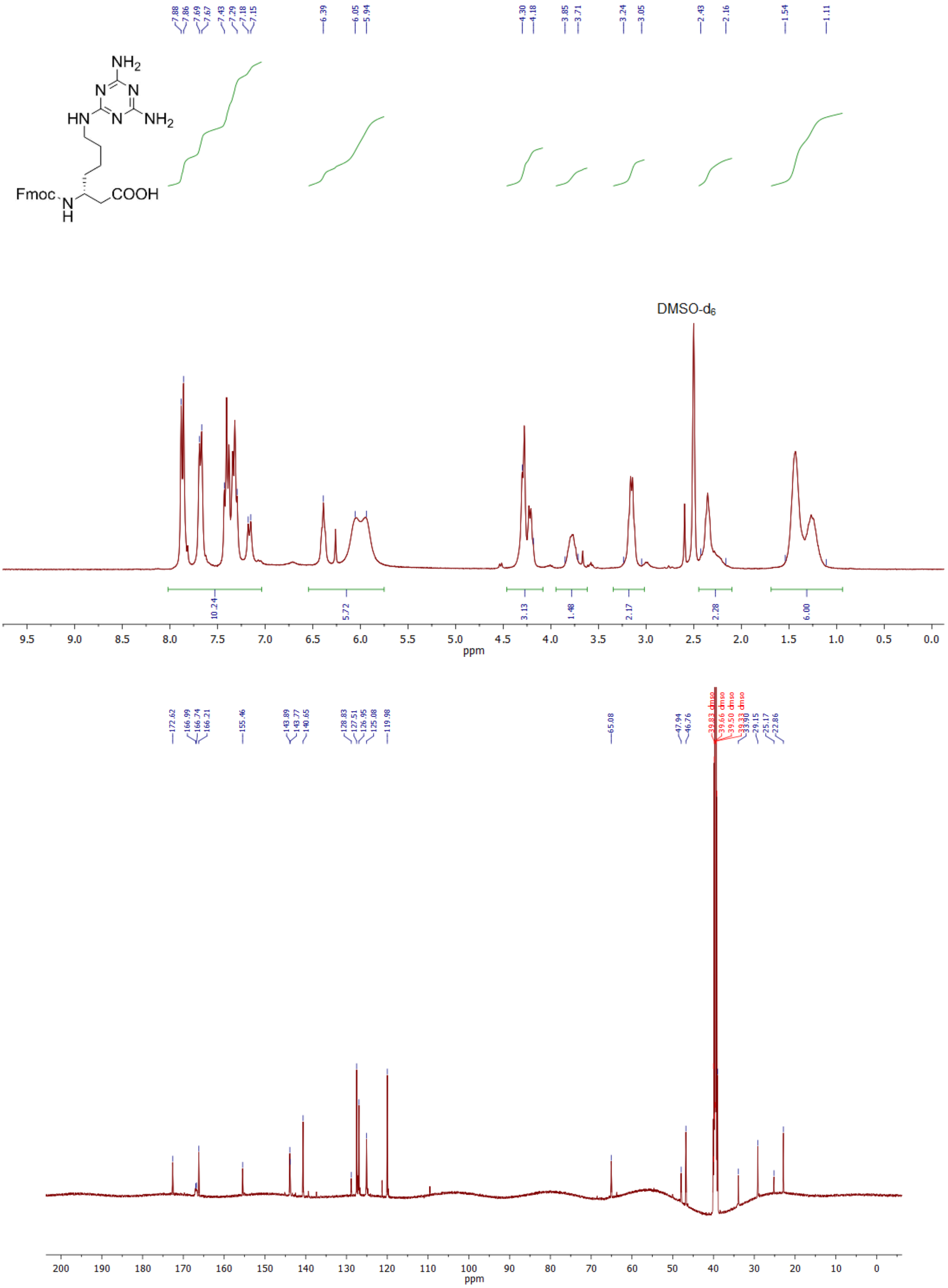

Figure S16. ^{1}H- (above) and ^{13}C-NMR (below) of (*R*)-3-((((9*H*-fluoren-9-yl)methoxy)carbonyl)amino)-7-((4,6-diamino-1,3,5-triazin-2-yl)amino)heptanoic acid, Fmoc-D-β^{3}-hLys(TAT)-OH (**12**).

5.2 HPLC-Chromatograms and ESI-HRMS Spectra of β^{3}-D-Peptides

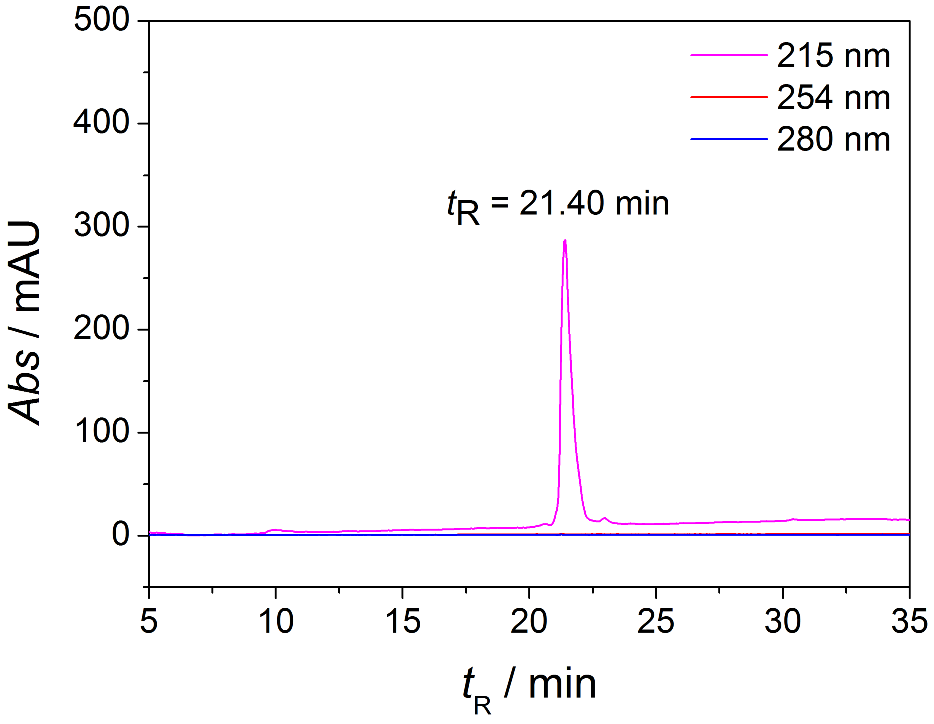

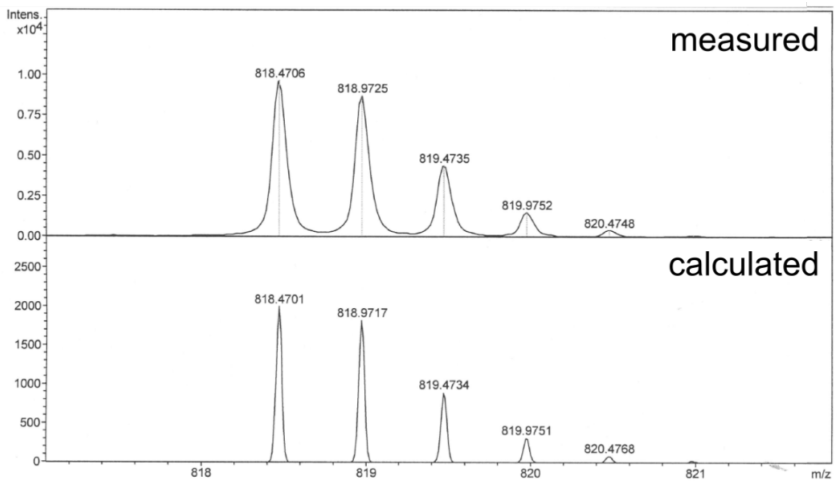

Figure S17. HPLC-chromatogram of β^{3}-CYA decapeptide **1** (top) and **B** ESI-HRMS spectra of peptide **1**, (*M* + 2H)$^{2+}$ selected as example (bottom).

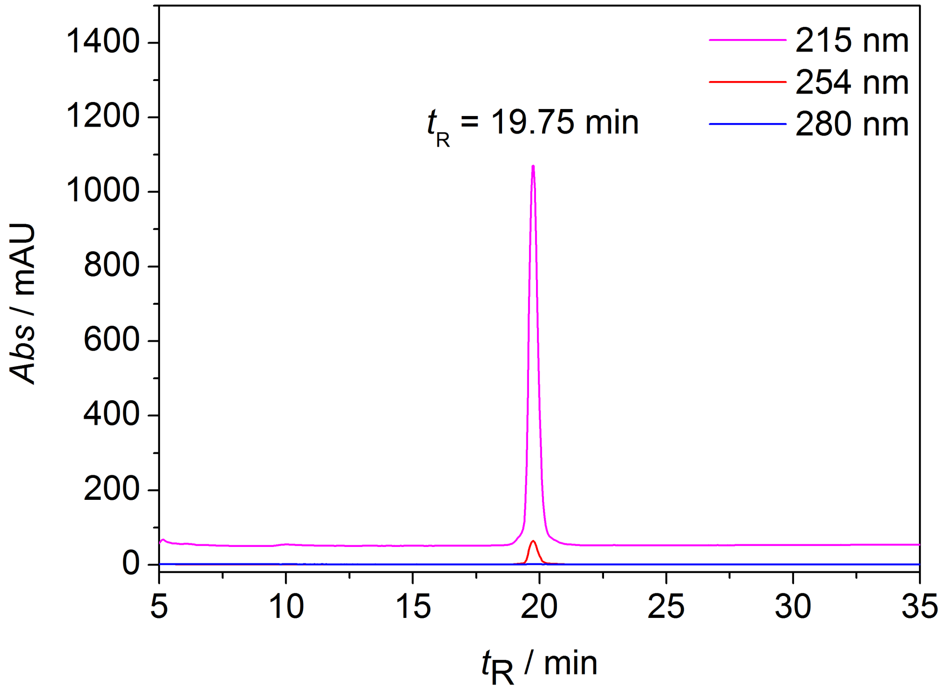

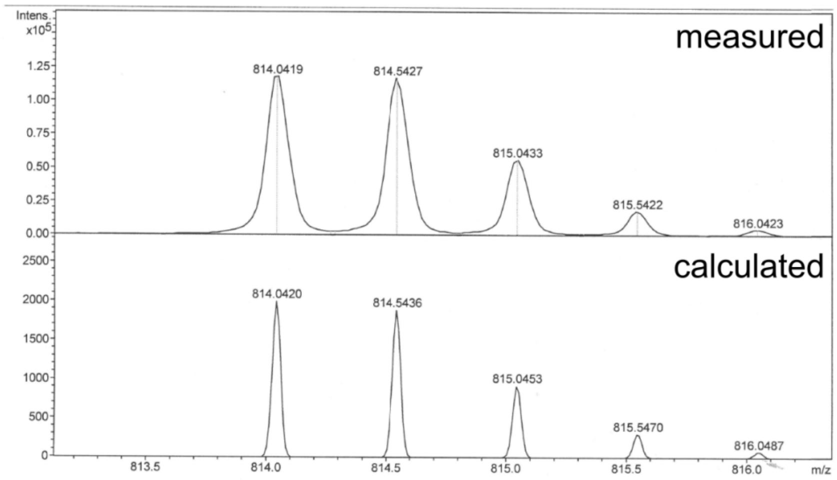

Figure S18. HPLC-chromatogram of β^3-TAT decapeptide **2** (top) and **B** ESI-HRMS spectra of peptide **2**, (M + 2H)$^{2+}$ selected as example (bottom).

6. References

[1] F. Arndt, C. R. Noller, I. Bergsteinsson, *Org. Synth.* **1935**, *15*, 3.
[2] (a) D. H. Appella, P. R. Leplae, T. L. Raguse, S. H. Gellman, *J. Org. Chem.* **2000**, *65*, 4766–4769. (b) M. Schinnerl, J. K. Murray, J. M. Langenhan, S. H. Gellman, *Eur. J. Org. Chem.* **2003**, 721–726.
[3] (a) D. Seebach, M. Overhand, F. N. M. Kühnle, B. Martinoni, L. Oberer, U. Hommel, H. Widmer, *Helv. Chim. Acta* **1996**, *79*, 913–941. (b) D. Seebach, P. E. Ciceri, M. Overhand, B. Jaun, D. Rigo, *Helv. Chim. Acta* **1996**, *79*, 2043–2066. (c) G. Guichard, S. Abele and D. Seebach, *Helv. Chim. Acta* **1998**, *81*, 187–206. (d) P. I. Arvidsson, M. Rueping, D. Seebach, *Chem. Commun.* **2001**, 649–650.
[4] (a) J. Wegner, G. Valora, K. Halbmair, A. Kehl, B. Worbs, M. Bennati, U. Diederichsen, *Chem. Eur. J.* **2019**, *25*, 2203–2207. (b) U. Rost, C. Steinem, U. Diederichsen, *Chem. Sci.* **2016**, *7*, 5900–5907.
[5] M. Gude, J. Ryf, P. D. White, *Lett. Pept. Sci.* **2002**, *9*, 203–206.
[6] J. K. Murray, S. H. Gellman, *Org. Lett.* **2005**, *7*, 1517–1520.
[7] R. C. Hirt, R. G. Schmitt, *Spectrochim. Acta* **1958**, *12*, 127–138.
[8] I. M. Klotz, T. Askounis, *J. Am. Chem. Soc.* **1947**, *69*, 801–803.
[9] Y. Zhang, A. A. Beckstead, Y. Hu, X. Piao, D. Bong, B. Kohler, *Molecules* **2016**, *21*, 1645.
[10] A. Wishard, B. C. Gibb, *Supramolecular Chemistry* **2019**, *9*, 608–615.
[11] M. Loos, C. Gerber, F. Corona, J. Hollender, H. Singer, *Anal. Chem.* **2015**, *87*, 5738–5744.

Author contribution

U. D. and S. K. G. conceived the project and wrote the manuscript through contributions of all authors. S. K. G. performed all chemistry work and sample preparations. K. K. performed ESI measurements of the aggregates. D. H. performed DLS measurements under the supervision of P. V. ESI measurements of preliminary compounds were performed by H. F. All authors have given approval to the final version of the manuscript.

Acknowledgements

We thank Brigitte Worbs for the synthesis of Fmoc-ACHC-OH, Daniel Frank for the production of diazomethane and *N*-nitroso-*N*-methylurea and the Deutsche Forschungsgemeinschaft (DFG) for generous support in context of the SFB 803 (project A01).
